# mJ-level 7-octave ultraflat white laser encompassing 200–25,000 nm

**DOI:** 10.1038/s41377-025-02142-z

**Published:** 2026-01-20

**Authors:** Lihong Hong, Renyu Feng, Yuanyuan Liu, Junming Liu, Junyu Qian, Yujie Peng, Yuxin Leng, Ruxin Li, Zhi-Yuan Li

**Affiliations:** 1 School of Physics and Optoelectronics, South China University of Technology, Guangzhou, 510641 China; 2 State Key Laboratory of Ultra-intense Laser Science and Technology, Shanghai Institute of Optics and Fine Mechanics, Chinese Academy of Sciences, Shanghai, 201800 China; 3 Guangdong Jingqi Laser Technology Corporation Limited, Dongguan, 523808 China; 4 State Key Laboratory of Luminescent Materials and Devices, South China University of Technology, Guangzhou, 510640 China

**Keywords:** Nonlinear optics, Supercontinuum generation, Ultrafast lasers

## Abstract

An intense ultrafast pulse white laser with continuous and ultraflat spectral coverage from deep-ultraviolet (DUV) to far-infrared (FIR) can open up a new arena of full-spectrum laser spectroscopy with applications to a wide variety of basic science and technology areas. Here, we present the creation of an intense white laser with 200–25,000 nm bandwidth @17 dB and ~1 mJ pulse energy by exploiting the synergic action of a high-efficiency nonlinear up-conversion module and down-conversion module upon an intense mid-infrared (MIR) seed pulse laser. The MIR seed pulse laser of 3.62 mJ pulse energy is achieved by sending an optical-parametric chirped pulse amplification pulse laser of 7.12 mJ pulse energy and 3.9 µm central wavelength through a krypton gas-filled hollow-core fiber. The up-conversion nonlinear module is a deliberately designed chirped-periodic poling lithium niobate (CPPLN) nonlinear crystal supporting simultaneous broadband second-order nonlinear 2nd–12th harmonic generation upon the seed laser to generate the shortest DUV wavelength down to 200 nm with a nearly 40% conversion efficiency. The down-conversion nonlinear module is composed of a bare LN crystal offering third-order nonlinear spectral broadening effect and a cascaded AgGaSe_2_ nonlinear crystal offering high-efficiency intra-pulse difference-frequency generation, and generates a 2000–25,000 nm MIR-FIR laser with an overall conversion efficiency of 18%. The intense 7-octave ultraflat DUV-FIR white laser would offer an unprecedented power to simultaneously probe and monitor the electronic transition, molecular vibration, and lattice oscillation in a wide variety of physical, chemical, and biological substances and processes.

## Introduction

Scientists have a long history of using various optical spectroscopy methods to probe, monitor, understand, and harness the microscopic and macroscopic physical, chemical, and biological world, as well as remote macroscopic cosmos^1,2^. In the late 1800s, observation and measurement of solar hydrogen absorption spectrum and discovery, in particular, of the hydrogen discrete absorption lines, helped to disclose the secrecy of the microscopic atom world, and set the foundation for the construction of quantum physics and mechanics^3–5^. In the late 1980s, the usage of the femtosecond pulse laser pump-probe technique helped to measure, evaluate, and disclose the secrecy of the molecular chemical bond breakage process and opened up the arena of femtosecond chemistry^6–8^. In the 1990s, the usage of attosecond pulse lasers enabled probing and monitoring electron motion within atoms, molecules, and solids^9–11^. Many, many other milestones, progresses, and achievements have been recorded in the history of optical spectroscopy. Classical optical spectroscopy uses a broadband natural or man-made incoherent light source like sunlight, a candle, or a halogen lamp, to serve as the illumination source^2^. The invention of the laser, a coherent light source with much better performance in spectral brightness, light beam collimation, and temporal resolution, has revolutionized optical spectroscopy and created various modern laser spectroscopy tools that have been broadly used in physics, chemistry, material science, biology, and information sciences^12–17^. In particular, the introduction of ultrafast pulse lasers into optical spectroscopy allows one to probe and monitor various ultrafast physical, chemical, and biological processes^13,14,18–20^.

There are many interesting physical, chemical, and biological phenomena and processes in atoms, molecules, solids, liquids, and cells that are of critical importance to deepen our understanding of the microscopic world and offer better solutions to numerous applications in our society and industry. Some prominent examples are light absorption and emission due to electronic state transition in atoms and molecules, formation and breakage of molecular bonds, inter-band transition in semiconductors, atomic vibrations and oscillations in small molecules and macromolecules, formations of phonons, plasmons, polarons, magnons and other electron/atom/ion group motion modes, molecular fingerprints and functional groups in proteins, lipids, and other biological substances, and so on^4,21,22^. In essence, all of them originate from the microscopic motions of electrons and atoms. Importantly, the characteristic energy scale of them varies across an extremely broad range of spectral windows from deep-ultraviolet (DUV), ultraviolet (UV), visible (Vis), near-infrared (NIR), mid-infrared (MIR), up to far-infrared (FIR), and even terahertz (THz) wavelengths. Notice that no individual incoherent thermal light sources, including sunlight, halogen lamp, and silicon-carbide elements lamp, exist that offer so a broad optical spectrum to probe so different microscopic processes simultaneously^23^.

It is highly desirable if one can develop a full-spectrum laser that covers an extremely broad spectral window, and moreover, has good coherence, high spectral brightness, and good directionality. It is even better that the full-spectrum laser has ultrashort pulse duration, intense pulse energy, and high-flatness spectra profiles. Optical spectroscopy constructed based on such a laser would exhibit unprecedented power for basic research, because many advantages can be expected. First, full-spectrum coverage enables direct probing of a wide variety of microscopic processes individually. Second, a coherent laser source with full-spectrum coverage allows monitoring the coherent quantum coupling of several microscopic processes with a big difference in energy scales (like electronic transitions in UV-Vis regimes and molecular vibrations in MIR regimes). Third, full-spectrum coverage allows direct use of the most sensitive absorption spectroscopy method rather than higher-order indirect spectroscopy methods (such as popular Raman spectroscopy). Fourth, ultrashort pulse duration enables probing and monitoring ultrafast processes or slow processes in an ultra-high temporal resolution. Fifth, intense pulse energy allows implementing and completing spectroscopy measurement by using only a single pulse, which would open up a new arena of high-speed spectrography (comparable with high-speed photography)^24^. Sixth, full-spectrum coverage with a high-flatness spectral profile helps to simultaneously probe a series of processes in different energy scales with high sensitivity and high fidelity. Finally, an ultrafast all-spectrum laser would bring more flexible operation modes of pump-probe ultrafast laser spectroscopy, ranging from narrow-band pump/narrow-band probe mode, to narrow-band pump/broadband probe and broadband pump/broadband probe modes.

Several schemes have been explored and implemented to develop and apply broadband lasers. The most popular scheme is the supercontinuum generation (SCG) enabled by various third-order nonlinear (3rd-NL) effects (like self-phase modulation (SPM)), stimulated Raman scattering, four-wave mixing, soliton fission, and dispersive-wave (DW) generation occurring in silica/tellurite/fluoride/chalcogenide glass photonic crystal fibers (PCF), hollow-core fibers (HCF), liquid-core optical fibers, gas-filled HCF, and other microstructured fibers upon the pico/femtosecond pump pulse laser^20,25–35^. These fiber supercontinuum lasers have been successfully demonstrated across various spectral bands from UV, Vis, NIR, to MIR, with each type of supercontinuum laser only covering a limited spectral range and altogether covering 100 nm to 18 μm^27–35^. Yet, due to a series of geometric and physical limitations, the pulse energy of a supercontinuum laser is very low (~nJ–${\rm{\mu }}{\rm{J}}$, « mJ). Besides, if the entire UV-Vis-NIR-MIR spectrum is concerned, the spectral flatness is limited, usually on the order of 30–60 dB in fluctuation^20,35^. An alternative scheme for spectral broadening is to utilize various second-order nonlinear (2nd-NL) interactions, including second-harmonic generation (SHG), sum-frequency generation (SFG), difference-frequency generation (DFG), and other three-wave mixing processes, in traditional bare or microstructured nonlinear crystals with either broadband birefringence phase matching (BPM) or broadband quasi-phase matching (QPM). These 2nd-NL effects, in principle, endorse an intensity several orders of magnitude larger than 3rd-NL interactions and thus can occur at a much lower peak power level of the pump. A recent study combining microstructured fibers with DFG nonlinear crystals has achieved a magnificent spectral coverage from 340 nm to 40 μm, albeit with limited flatness (−70 dB) and modest pulse energies (~0.45 μm)^23^. Promisingly, a series of high-harmonic generation (HHG, achieved via cascaded harmonic generation through ${\chi }^{(2)}$ three-wave mixing processes) processes in synergy with 3rd-NL processes can be explored to encompass an extremely broadband and flat spectrum^36–38^. Within this framework, recently, a full-spectrum white laser with a 25 dB bandwidth of 300–5000 nm and a 0.54 mJ pulse energy was realized by sending a 3.3 mJ MIR optical-parametric chirped pulse amplification (OPCPA) femtosecond pulse laser centered at 3.9 µm through a cascaded architecture of gas-filled HCF and bare lithium niobate (LN) crystal offering two-fold 3rd-NL spectral broadening, and a chirped periodically poled lithium niobate (CPPLN) bulk crystal that offers multiple broadband QPM bands for 2nd-10th HHG processes and endures a very high pump power up to 10 mJ^39^.

In this work, we promote the OPCPA MIR pulse energy to a maximum of 7.12 mJ and use it to pump a krypton (Kr) gas-filled HCF to create an intense MIR seed pulse laser with a broad bandwidth of 2000–6000 nm and large pulse energy of 3.62 mJ. This seed laser then serves as a middle bridge to pump a nonlinear up-conversion module and a nonlinear down-conversion module for creating an intense full-spectrum ultrafast white laser. The up-conversion module consists of a newly designed and better-performed CPPLN crystal to trigger 2nd–12th HHG upon the intense MIR seed pulse laser, leading to a white laser with spectral range 200–6000 nm (nearly 5-cotave) and maximum pulse energy 1.45 mJ. The down-conversion module consists of a bare LN crystal igniting further 3rd-NL spectral broadening and a cascaded AgGaSe_2_ (AGSe) nonlinear crystal offering high-efficiency intra-pulse DFG (IP-DFG) upon the significantly broadened MIR seed pulse laser, leading to an extremely broad bandwidth 2000–25,000 nm and an intense pulse energy 0.75 mJ. This nonlinear up-conversion and down-conversion synergy strategy allows one to use the mediate ~3.9 µm MIR intense pulse laser for creating mJ-level intense 7-octave full-spectrum ultrafast white laser with ultraflat spectral profile. This simple scheme can pave the way toward building a tabletop full-spectrum laser that is armed with a series of unique merits, including extremely broadband spectral coverage, intense pulse energy, high spectral flatness, ultrafast pulse duration, and good coherence. This white laser surely will revolutionize laser technology and adjunct laser spectroscopy, which would open up a very broad arena of basic science and high technology applications.

## Results

### Principle of full-spectrum white laser generation

**Fig. 1:**
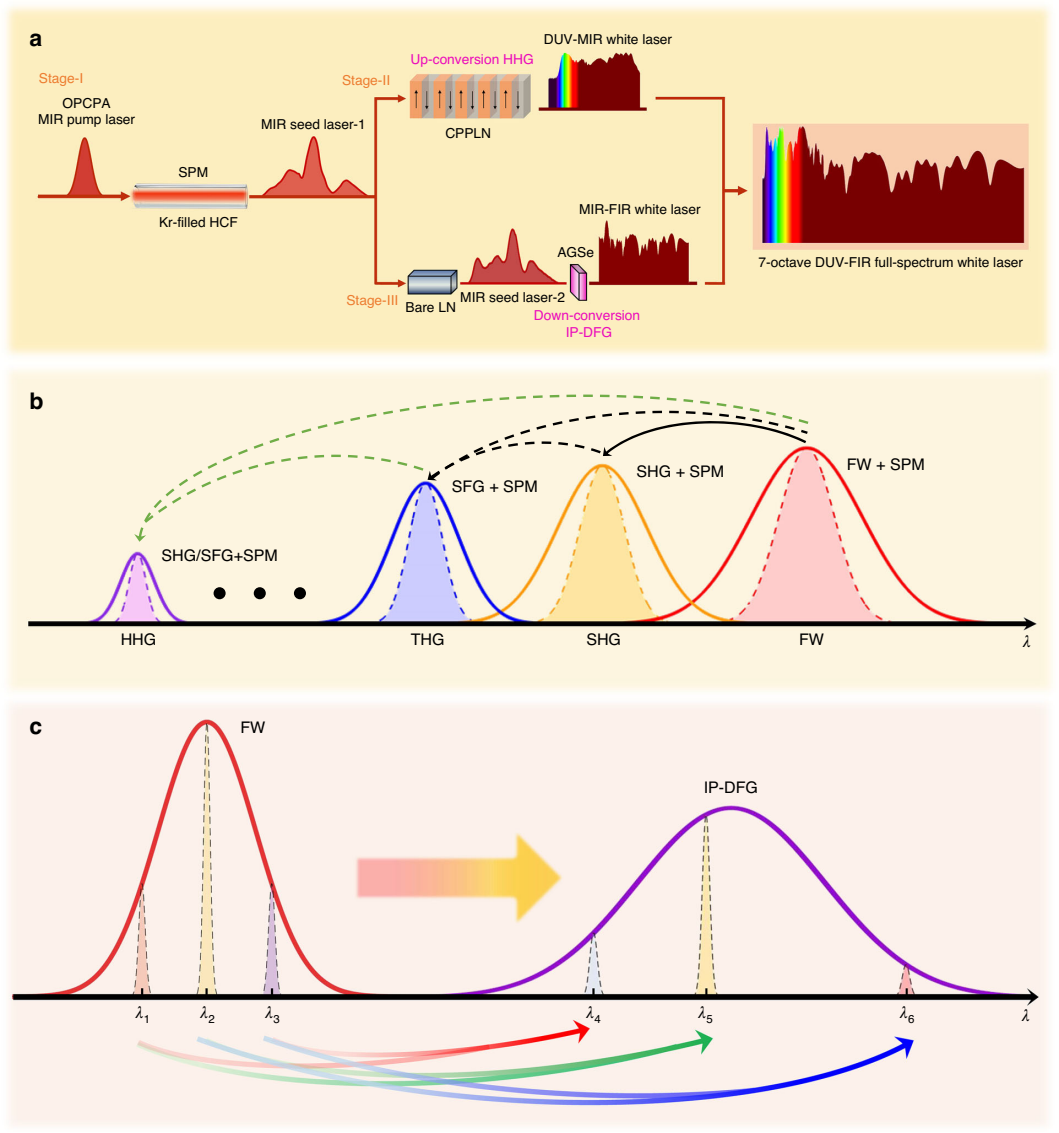
Principle for nonlinear up-conversion and down-conversion synergy strategy toward creating an intense full-spectrum white laser. **a** The full-chain process diagram of the full-spectrum white laser generation. Stage I, Schematic diagram for sending an intense OPCPA MIR pump laser through a Kr-gas-filled HCF to create an intense MIR seed pulse laser with broad bandwidth and large pulse energy. This seed laser then serves as a middle bridge to pump a nonlinear up-conversion module and a nonlinear down-conversion module for creating a full-spectrum intense pulse white laser with spectral coverage from DUV to FIR. Stage II, Schematic diagram for nonlinear up-conversion upon an intense seed MIR pulse laser passing through the CPPLN nonlinear crystal to trigger 2nd–12th HHG. Stage III, Schematic diagram for nonlinear down-conversion upon an intense MIR seed pulse laser that passes through a cascaded module of a bare LN crystal for triggering broader 3rd-NL spectral broadening and AGSe nonlinear crystal for igniting IP-DFG, and greatly expands the long-wavelength end of the MIR spectrum down to FIR. **b** Schematic of ultrabroadband 2nd–12th HHG processes involving a series of cascaded three-wave mixing processes accompanied by SPM effect. **c** Schematic of the generation of down-conversion radiation via IP-DFG processes

Realizing a full-spectrum white laser spanning from DUV to FIR requires precise control of nonlinear optical processes and material properties under intense pump laser excitation while maintaining maximal bandwidth. This achievement relies on two critical advancements: (1) the development of novel nonlinear media with exceptional broadband phase-matching capabilities and (2) optimized pump-scheme configurations, such as octave-spanning femtosecond lasers, to extend supercontinuum generation into the entire DUV-FIR regime. The operation principle is schematically depicted in Fig. 1a, where a broadband MIR seed source originating from an intense OPCPA MIR pump laser undergoes both up-conversion and down-conversion within nonlinear crystals. The diagram highlights how tailored phase matching and pump-power management enable efficient wavelength expansion across the extreme DUV-FIR spectrum, thereby realizing the theoretical framework experimentally.

Specifically, the system comprises three key parts separated into three stages. The initial part (Stage I) employs a 3-meter-long Kr-filled HCF to spectrally broaden a 3.9 μm MIR pump laser from a home-built OPCPA system via SPM. This initial stage generates a MIR supercontinuum with octave-spanning coverage, providing ideal driver wavelengths for subsequent nonlinear frequency conversion processes. For the second part (Stage II), a longitudinally chirped CPPLN crystal is integrated to shift the MIR spectrum toward shorter wavelengths through high-efficiency up-conversion HHG. The engineered chirped structure of CPPLN enables multi-stage efficient three-wave mixing processes across a broadband pump range, which significantly enhance the spectral density and flatness in the DUV-MIR region. The final part (Stage III) utilizes a cascaded arrangement of bare LN (offering 3rd-NL spectral broadening) and AGSe nonlinear crystals (exhibiting excellent 2nd-NL IP-DFG) to drive efficient frequency down-conversion from the MIR pump field into the FIR regime. This dual-crystal configuration leverages their complementary 2nd and 3rd nonlinear properties to achieve smooth and broadband down conversion without introducing significant loss or distortion. The synergic integration of these three physical mechanisms—3rd-NL spectral broadening, UV-enhanced 2nd-NL up-conversion, and FIR-efficient 2nd-NL down-conversion—ultimately realizes the long-standing goal of generating an intense, high-flux, and flat-topped full-spectrum ultrafast white laser across the DUV-FIR continuum.

The ultrabroadband HHG process in the specially designed CPPLN crystal with broadband reciprocal lattice vector (RLV) bands relies on a cascade of 2nd-NL nonlinear three-wave mixing and synergic 3rd-NL SPM effect of each harmonic. As illustrated in Fig. 1b, the intense fundamental-wave (FW) MIR femtosecond pulse laser first initiates multiple second-order nonlinear interactions, with cascaded SHG and SFG effects emerging as dominant pathways under ultrabroadband QPM conditions. Specifically, the FW pulse undergoes SHG to generate an SHG pulse, which then interacts with residual FW via SFG to produce a THG pulse. Both harmonic pulses propagate coherently with the FW pulse, forming a multi-wavelength pump field that efficiently ignites HHG, as SHG and THG can now become the starting point to create other higher-order nonlinear up-conversion processes with the short wavelength down to DUV, consistent with the theoretical framework established in ref. ^39^. When the conversion efficiency is high enough, these HHG pulses still have high enough intensity to ignite 3rd-NL effects like SPM to further broaden their own spectral bandwidth and enable different harmonics to spectrally connect to each other. Ultimately, the synergic integration of 2nd- and 3rd-order nonlinear processes yields a DUV-MIR supercontinuum white laser with exceptional spectral quality by gap-free connection of the pump and HHG waves.

The IP-DFG process in nonlinear crystals with excellent MIR performance (high transparency and nonlinearity) is systematically investigated using a tri-component pump scheme, as illustrated in Fig. 1c. By decomposing the broadband input laser into three randomly selected narrow-band wavelengths (e.g., ${\lambda }_{1}$ = 3 μm, ${\lambda }_{2}$ = 4 μm, and ${\lambda }_{3}$ = 5 μm), we isolate distinct frequency-mixing pathways by exploiting BPM condition across a broadband pumping. The interaction between two widely separated pump pulses (e.g., the shortest ${\lambda }_{1}$ = 3 μm and longest ${\lambda }_{3}$ = 5 μm) generates a short-wavelength DFG beam (${\lambda }_{4}$ = 7.5 μm). Meanwhile, when two closely spaced pumps (e.g., ${\lambda }_{1}$ = 3 μm and ${\lambda }_{2}$ = 4 μm) co-propagate, their nonlinear mixing initially produces a long-wavelength DFG signal (${\lambda }_{5}$ = 12 μm). These two pumping strategies effectively fill the low-frequency gap between the MIR and FIR regions. Furthermore, DFG between two adjacent pulses with longer components (e.g., ${\lambda }_{2}$ = 4 μm and ${\lambda }_{3}$ = 5 μm) would generate a longest-wavelength spectrum (${\lambda }_{6}$ = 20 µm), filling in the longer wave portion of the MIR-FIR supercontinuum. Overall, for a broadband driving pulse, the synthetic multiple-channel IP-DFG scheme is effectively supported by the broadband BPM of a high-performance DFG nonlinear crystal and eventually works to accumulate with high efficiency the generation of a multi-octave infrared spectrum ranging from MIR to FIR.

### OPCPA upscaling and experiment layout

**Fig. 2:**
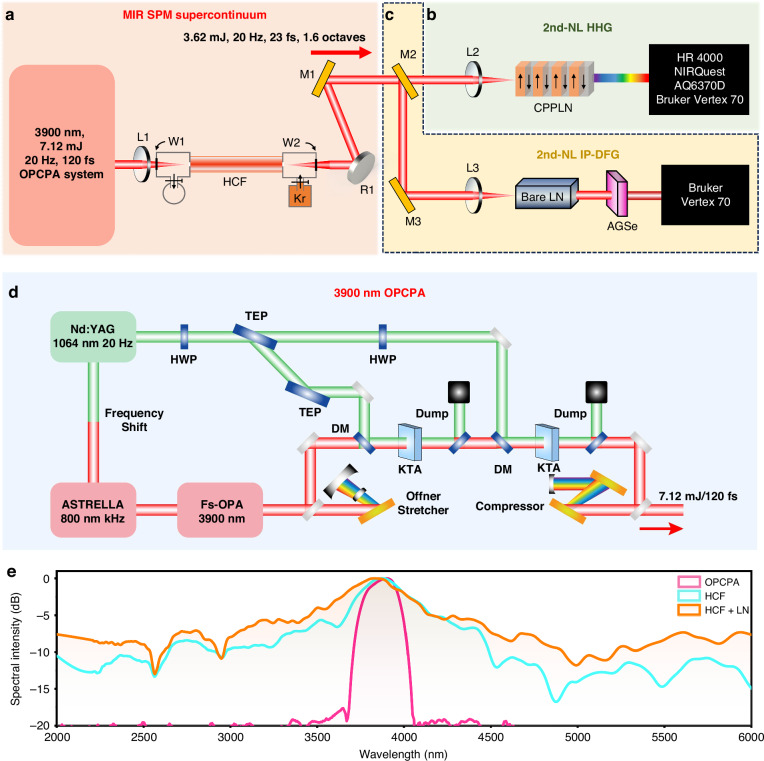
Schematic of the system layout for generating a high-flatness full-spectrum white laser. **a** Generation of intense broadband MIR seed pulse laser. By sending an intense OPCPA femtosecond pump laser with a central wavelength of 3.9 ${\rm{\mu }}{\rm{m}}$ and pulse energy up to 7.12 mJ, through a Kr-gas-filled HCF, an intense broadband MIR seed pulse laser is obtained with 2000–6000 nm bandwidth @ 17 dB and up to 3.62 mJ pulse energy. L lens, W CaF_2_ window, M Au-coated mirror, R concave mirror, Kr Krypton. **b** Generation of intense DUV-MIR pulse white laser via nonlinear up-conversion upon the broadband MIR seed pulse laser passing through a specially designed CPPLN crystal with multiple superior broadband RLV bands offering multiple high-efficiency HHG processes. **c** Generation of intense MIR-FIR pulse laser via nonlinear down-conversion upon the broadband MIR seed pulse laser passing through a cascaded module consisting of a bare LN crystal, igniting further 3rd-NL spectral broadening to build a smoother second-stage MIR supercontinuum seed laser, and subsequently delivering into an AGSe nonlinear crystal offering high-efficiency IP-DFG. **d** Schematic of the 3.9 µm OPCPA system. **e** Measured spectra for the intense OPCPA pump pulse laser and the resulting two-stage intense MIR seed pulse lasers from the HCF and HCF-LN cascaded module, respectively. All spectra are normalized against their maximum values

We describe the important details of our experiment in Fig. 2. As depicted in Fig. 2a, firstly, an upgraded home-built OPCPA system serves as the pump laser source for the subsequent nonlinear spectral expansion HCF setup. The schematic optical layout of the 3.9 μm OPCPA intense laser system with 7.12 mJ is presented in Fig. 2d^40,41^. The system employs a multi-component setup for efficient MIR pulse generation, involving a 1 kHz Ti: sapphire chirped pulses amplification (CPA) laser, a KTiOAsO_4_ (KTA) crystal-based fs-OPA seed laser scheme, an Öffner stretcher, a two-stage picosecond OPA, a specially designed Nd: YAG picosecond pump laser, and a grating-pair compressor. The 1 kHz Ti: sapphire CPA laser generates 36 fs, 3 mJ pulses at 800 nm, which serve as the pump for a KTA-based femtosecond OPA to generate the seed for the following OPCPA. This KTA-based OPA uses type II phase-matching (*θ* = 38.3°) to generate a 120 μJ seed pulse at 3.9 μm. To match the pump duration of the subsequent picosecond OPA, the seed pulse is stretched to 70 ps using a conventional Öffner stretcher with a 300 grooves/mm gold-coated grating, achieving 37.5% efficiency and yielding a 45 μJ stretched pulse. The Nd: YAG pump laser (20 Hz, 70 ps, 1064 nm, up to 250 mJ) for the OPCPA is synchronized with the Ti: sapphire system via wavelength shifting and split into two beams (30 mJ/Φ3 mm and 180 mJ/Φ6 mm). These beams are then transmitted into the first and second crystals by image relaying. In order to improve the amplifier efficiency, the flat-top beam profile is used to provide a spatially uniform parametric gain for the signal pulse.

The picosecond OPA system utilizes two KTA crystals (type II phase-matching, *θ* = 40.8°) with lengths of 10 mm and 16 mm for two-stage amplification. In the first OPA stage, a 4f optical system reduces the beam sizes of the signal and pump pulses to 3 mm, enabling efficient collinear injection into the 10 mm KTA crystal. With a pump intensity of 6.06 GW/cm^2^, the signal pulse is amplified to 1.1 mJ and retained for further amplification. To protect components from damage by high-intensity pump pulses, beam sizes are expanded to 6 mm in the second stage, increasing the pump intensity to 9.1 GW cm^−2^ and boosting the signal energy to 11.4 mJ. Then, the amplified signal pulse after the second OPA stage is transmitted into a grating-pair compressor. This compressor is specifically engineered to conjugate the stretcher for accurate dispersion compensation. After being compressed by the grating-pair compressor, the energy of the amplified 3.9 μm pulses is 7.12 mJ. Through optimization of the grating angles and separation distance within the compressor, the pulse achieves its shortest duration of 120 fs, which is close to the Fourier transform limit duration of 105 fs. This output spectral curve is shown by the pink line in Fig. 2e, spanning the range of 3500–4050 nm.

Subsequently, the initial intense MIR pump pulse undergoes remarkable spectral broadening via a remarkable SPM effect when injected into a Kr-filled HCF with 1 mm inner-core diameter and 3 m length by an *f* = 800 mm CaF_2_ lens^40–43^. The nonlinear interaction characteristics are controlled through Kr pressure *p* optimization (in units of bar), with nonlinear refractive index *n*_2_ = 2.7*p* × 10^−23^m^2^ W^−1^. We design the Kr at pressures up to 2.2 bar to achieve an optimum broadened MIR supercontinuum spectrum spanning 2000–6000 nm with 4.04 mJ of pulse energy. Then, a 2-mm-thick CaF_2_ plate, which can introduce a certain amount of negative dispersion, is used for recompressing the pulse to ~ 23 fs, and a concave mirror with *R* = 600 mm is used for collimating the output light with a total efficiency of 51% and 3.62 mJ of output pulse energy. This process creates a fresh MIR seed pulse laser with supercontinuum spanning from 2000 nm to 6000 nm at the −17 dB level (the cyan line in Fig. 2e) for the following two-stage 2nd-NL interactions. Systematic characterization of beam stability and spatial profiles confirms exceptional performance with OPCPA stability of 2.29% root mean square (RMS) and HCF-mediated beam quality improvement, providing the robust foundation for the white-light generation (see Supplementary Note 1 for details).

The up-conversion HHG setup is schematically shown in Fig. 2b. The collimated output MIR seed laser from the HCF setup is directed into a 2-cm-length CPPLN nonlinear crystal by an *f* = 200 mm CaF_2_ lens (with ~2 mm beam diameter at the crystal entrance) to trigger high-efficiency and ultrabroadband HHG. A combination of optical spectrum analyzers is employed to measure the DUV-Vis-MIR spectra. For the down-conversion IP-DFG process, we use two Au-plated mirrors to redirect the output MIR supercontinuum beam to another separate experimental pathway, as shown in Fig. 2c. Here, the MIR supercontinuum laser beam is first focused into a 2-cm-length bare LN bulk crystal through an *f* = 200 mm CaF_2_ lens to induce a further dramatic 3rd-NL induced spectral broadening. As demonstrated by the orange curve in Fig. 2e, this process significantly improves spectral flatness (~4 dB enhancement) and extends the continuum coverage to 2000–6000 nm (13 dB bandwidth) with an output energy of 2.03 mJ. Theoretical analysis reveals that spectral broadening in the HCF is dominated by SPM in the normal dispersion regime, whereas the LN crystal operates in the anomalous dispersion regime and supports complex soliton dynamics and DW for spectral broadening (see Supplementary Note 2 for details). This smoother octave-spanning pulse laser serves as an optimal pump source for creating a high-quality ultrabroadband MIR-FIR white laser. Next, the high-flatness 1.6-octave MIR pulse laser is then collimated and vertically incident on the DFG crystal AGSe with a diameter of around 5 mm at the point of incidence to activate the nonlinear down-conversion scheme. In the same manner, the resultant white laser beam is characterized by a Fourier transform infrared (FTIR) optical spectrometer to make an accurate spectral analysis.

### DUV-MIR white laser generation upon up-conversion of HHG

**Fig. 3:**
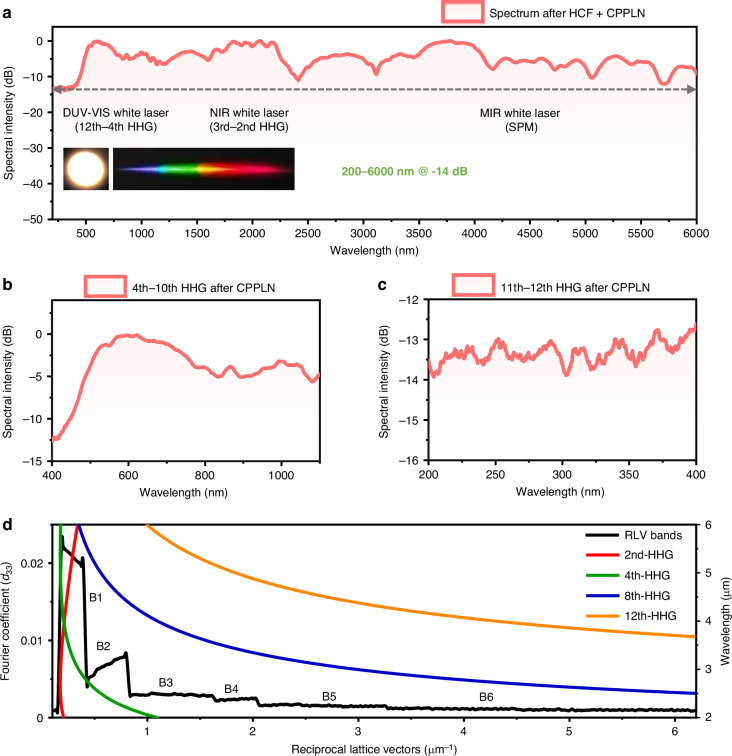
Measurement results of the DUV-MIR white laser from the HCF-CPPLN cascaded module. **a** Measured spectrum for up-conversion white laser showing 200–6000 nm @ 14 dB ultraflat spectral profile (nearly 5-octave) and a maximum pulse energy of 1.45 mJ (with a large conversion efficiency of 40%). The inset shows a photograph of the generated white-light spot and the dispersed visible spectrum of the output white laser. **b**, **c** The enlarged view of the 4th–12th HHG spectra. **d** A composite plot of Fourier coefficient curves in bands B1–B6 from the CPPLN sample superimposed with phase-mismatch curves for multiple up-conversion processes (e.g., 2nd-, 4th, 8th, and 12th-HHG) in a homogeneous LN crystal. B1–B6 are located at [0.14, 0.43], [0.43, 0.84], [0.84, 1.67], [1.67, 2.07], [2.07, 3.29], and [3.29, 6.28], respectively, all in units of μm^−1^

Figure 3a showcases the supercontinuum white laser spectrum, spanning 200–6000 nm at a 14 dB bandwidth, obtained from the CPPLN sample driven by the intense 1.6-octave MIR femtosecond pumping. The output spectrum ensures gap-free coverage across five critical spectral regions (DUV, UV, Vis, NIR, MIR), overcoming the limitations of traditional laser sources that often exhibit absorption losses or nonlinear saturation in specific bands. Such a superior 5 octave-spanning continuum arises from the wavelength-dependent dominance of different HHG orders. Specifically, the SHG (or 2nd-HHG) process generates distinct shoulder features at 2836 nm, 2057 nm, and 1821 nm, forming a broadband continuum from 1200 nm to 3000 nm. The THG (or 3rd-HHG) signal features shoulder peaks at 1167 nm and 1440 nm, spanning 666–2000 nm. The resultant emission profile of the 2nd- and 3rd-HHG signals forms a stable white source in the NIR region by bridging their respective continua. The 4th–12th HHG signals dominate shorter wavelengths, producing a DUV-VIS-NIR continuum from 200 nm to 1200 nm with discrete peaks at 994 nm, 865 nm, 666 nm, 623 nm, 537 nm, 486 nm, 431 nm, 371 nm, and 251 nm, alongside associated shoulder structures, as shown in Fig. 3b, c. These broadband HHG signals overlap and merge with the FW pump signal, resulting in a gap-free DUV-MIR spectrum from 200 to 6000 nm at a 14 dB intensity level, with an estimated pulse width of approximately 1.8 ps by numerical simulations (see Supplementary Note 3 for details). Numerical simulations also show that although the DUV spectral components (200–280 nm) due to 12th-HHG are located in the LN material absorption window, the absorptions are not a serious issue because they occur only at a very short length before the crystal exit end.

The output laser energy after CPPLN reaches a high level of up to 1.45 mJ per pulse with an overall conversion efficiency of ~40%. At this power level, the spectral energy of DUV band (200–248 nm), the UV band (248-380 nm), the Vis band (380–780 nm), the NIR band (780–2500 nm), and the MIR band (2500–6000 nm) is estimated to be 0.014 mJ, 0.024 mJ, 0.45 mJ, 0.36 mJ, and 0.35 mJ, respectively. Such a multi-octave, energy-efficient continuum not only minimizes spectral gaps but also delivers balanced power distribution across critical wavelength ranges, making it suitable for applications requiring full-spectrum high-spectral-density light sources. The insets in Fig. 3a show the generated naked-eye white-light spot and broadband visible spectrum of the collimated white laser output via diffraction grating dispersion, demonstrating both the excellent preservation of the original MIR pump beam geometry and broadband nature of the output white laser.

Our experimental realization highlights a paradigmatic integration of an innovative HCF-CPPLN 2nd-3rd-NL synergy technologies to achieve a powerful DUV-MIR white laser. The outstanding ultrabroad QPM capacity of the CPPLN sample, across an octave-spanning pump, is the key prerequisite for high-efficiency ultrabroadband and uniform HHG nonlinear up-conversion via various cascaded three-wave mixing processes (~36 cascaded SHG and SFG interactions)^39^. This is achieved through a sophisticated poling pattern design of the CPPLN sample, which elaborately harnesses multiple QPM bands (B1–B6) to address phase-mismatching challenges in HHGs against an octave-spanning MIR seed laser.

As the core element of the nonlinear up-conversion module, the newly designed and better-performed CPPLN crystal deliberately matches with the parameters of the current high-energy OPCPA MIR seed pump laser for triggering high-efficiency 2nd–12th HHG, and shows a much better performance compared with that used in ref. ^39^. (see Supplementary Note 4 for details). As displayed in Fig. 3d, band B1 covers the phase-matching curve of the 2nd-HHG, while bands B1–B3 predominantly compensate for the 4th-HHG process. Bands B2–B6 further extend the QPM coverage to enable efficient 8th–12th HHG processes. Similarly, the residual phase mismatches governing various 2nd–12th HHG processes are efficiently compensated for by the respective RLV bands of the engineered CPPLN sample.

### MIR-FIR white laser generation upon down-conversion IP-DFG

**Fig. 4:**
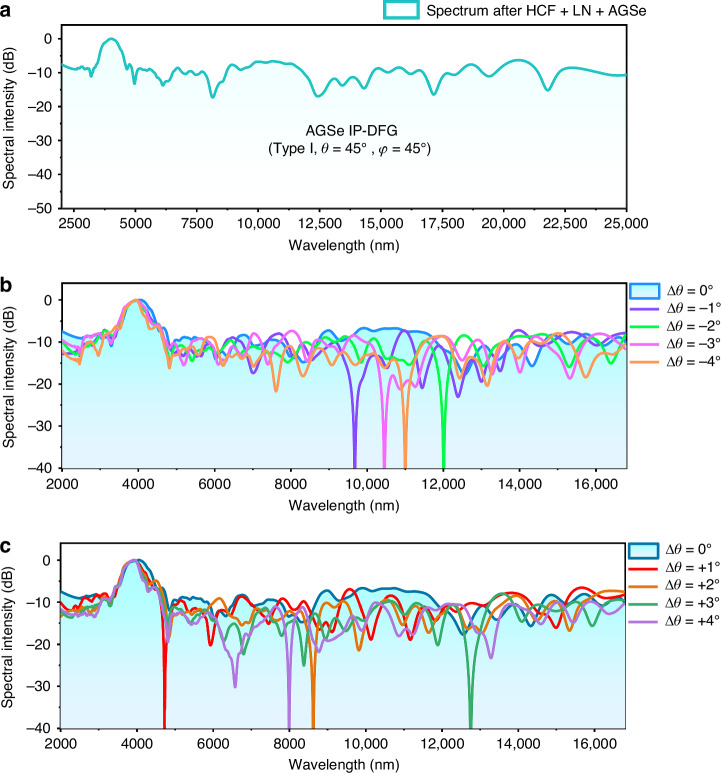
MIR-FIR supercontinuum laser from cascaded HCF-LN-AGSe module via nonlinear down-conversion IP-DFG. **a** Measured spectrum for the final down-conversion white laser output from the AGSe nonlinear crystal meeting 45° phase matching, showing 2000–25,000 nm @ 17 dB ultraflat spectral profile and a maximum pulse energy of 0.75 mJ (with a very large down-conversion efficiency ~18%). **b**, **c** Measured spectra for the output down-conversion laser from the AGSe nonlinear crystal with different orientation angles. The spectra of the output down-conversion laser from the AGSe in the case of 45° (i.e., $\Delta \theta ={0}^{\circ }$) phase matching is also illustrated as a reference

The MIR-FIR field generation via IP-DFG down-conversion is accomplished using a 1-mm thick AGSe crystal, selected for its minimal spatial walk-off, precise orientation control during growth and cutting processes, and compatibility with antireflection coatings^44^. The selected AGSe sample is antireflection (AR) coated, and cut at type I phase matching (eoo-branch) with polar angle *θ* = 45° and azimuthal angle *ϕ* = 45° for the normal incidence of the laser beam. To satisfy this phase-matching condition, one has to provide both ordinary (o) and extraordinary (e) polarized components of the pump electric field. For this purpose, we orient the crystal such that the polarization vector of the pump electric field has approximately equal o and e components. The IP-DFG spectrum obtained from the AGSe sample (type I phase matching, *θ* = 45°, *ϕ* = 45°, as designed) is presented in Fig. 4a. The obtained spectrum spans over 3.6 octaves, 2000–25,000 nm when measured at −17 dB level with respect to the maximum. Notice the FTIR spectrometer used has an upper response limit of 25,000 nm; the flat and high spectral intensity around 25,000 nm without showing a decaying trend strongly suggests that the infrared signal can go beyond 25,000 nm and well into the FIR regime, e.g., 30,000 nm, with considerable spectral energy and intensity. Notably, the maximum IP-DFG output energy exceeding 6000 nm is recorded as 0.26 mJ, achieved with a driving laser pulse energy of 1.45 mJ from the HCF-LN module, yielding an overall conversion efficiency of approximately 18%.

Some large dips are found in the IP-DFG spectrum, which are mainly caused by the spectral shapes in the short-wavelength and long-wavelength regions of the driving pulse. For example, the two dips at 3.28 µm and 5.49 µm in the pump spectrum (see the orange line in Fig. 2e) manifest to directly cause the IP-DFG dip at 8.15 µm as presented in Fig. 4a. Similarly, a long-wavelength idler dip at 21.66 µm arises from weak signal components of 2.57 µm and 2.95 µm in the driving pulse. These observations highlight the critical role of the spectral coherence, intensity, overlap, and flatness of the MIR seed pump laser in determining the IP-DFG output characteristics. Despite the observed spectral dips, this scheme exhibits exceptionally excellent integrated performance, achieving balanced spectral intensity distribution (up to 0.75 mJ output energy) across a 3.6-octave ultra-wide bandwidth, maintaining superior spectral flatness (~±5 dB variation), and delivering high overall IP-DFG conversion efficiency (~18%). To understand the underlying physics governing the highly efficient IP-DFG dynamics within the AGSe crystal, we perform a comprehensive theoretical analysis (see Supplementary Note 5 for details), which agrees well with experiment. These theoretical and experimental findings collectively establish a new paradigm for efficient MIR-FIR frequency conversion with low-loss spectral characteristics.

Furthermore, we delve into exploring the robustness of this IP-DFG scheme by making slight rotations of the AGSe crystal around the optimal matching angle. We meticulously adjust the orientation of the AGSe sample by making rotations around the optimal phase-matching angle *θ* = 45° (i.e., $\Delta \theta ={0}^{\circ }$) with $\Delta \theta =\pm {1}^{\circ } \sim \pm {4}^{\circ }$ and measure the corresponding MIR-FIR spectra, which are displayed in Fig. 4b, c. The IP-DFG MIR-FIR spectra exhibit both tunability and robustness over an average bandwidth of 2000 nm–25,000 nm at a stable level of around 20 dB. An imperfect thing is that some noticeable spectral irregularities arise from the phase-mismatching of AGSe. One can find that using smaller phase-matching angle (i.e., the polar angle $\theta ={44}^{\circ } \sim {41}^{\circ }$) cause long-wavelength flatness degradation, primarily concentrated in the 9.1–12.5 μm region (Fig. 4b). While increasing phase-matching angle (i.e., the polar angle $\theta ={46}^{\circ } \sim {49}^{\circ }$), the shorter-wave part covering 4.5–8.9 μm shows more dispersed spectral depressions, whereas the longer-wavelength section of 9–12.3 μm exhibits a relatively smoother flatness (Fig. 4c). These observations reveal that the generation of ultrabroadband IP-DFG has a significant tolerance to angular misalignment as manifesting in the robustness of the output signal upon a broadband driving pump. Furthermore, the output spectra can be subtly adjusted to accommodate various performance requirements for DFG spectral evolution.

The quadratic dependence of IP-DFG efficiency on the effective interaction length (*L*_eff_) fundamentally limits the performance of the nonlinear crystal, primarily due to group velocity mismatch between the pump and MIR pulses. This constraint narrows down when using longer-wavelength pump sources and shorter crystal length. In our experimental setup, assuming IP-DFG center wavelength ~10 µm and pulse duration of ~100 fs, we observe a marked increase in *L*_eff_ from 90 μm for a regular 1 μm pumping to 1.3 mm when employing a 3.9 μm pump source. This wavelength-dependent behavior arises because the 3.9 μm pump closely matches the MIR output wavelength of the idler beam generated via IP-DFG, thereby minimizing the group velocity dispersion. Such strategic pump wavelength selection not only achieves a 200-fold enhancement in IP-DFG conversion efficiency but also dramatically reduces the parasitic effects arising from multi-photon absorption. On the other hand, a broadband pump spectrum enables efficient energy transfer and spectral flattening in IP-DFG by an excellent phase-matching scenario over a wide wavelength range. This allows multiple pump frequency components to coherently contribute to down-conversion, significantly increasing the total energy and spectral intensity of the generated IR signal. The highly uniform distributed pump power across numerous channels also minimizes intensity variations, ensuring uniform energy distribution and suppressing nonlinear distortion mechanisms that preferentially affect narrow-band signals. Consequently, our ultrabroadband long-wavelength pumping approach greatly helps to achieve high-energy, octave-spanning MIR-FIR white laser generation with exceptional spectral flatness.

Our generated IP-DFG spectrum from the LN-AGSe module demonstrates exceptional broadband characteristics, spanning 3.6 octaves (2000–25,000 nm) with a 17 dB bandwidth, significantly surpassing most AGSe-based systems^44–46^. While Novák et al. achieved a relatively limited octave span of ~1.1 octaves (7000–15,000 nm) with a conversion efficiency of 0.8% using AGSe pumped by a 250 µJ, 2 µm source^46^, our work substantially extends the coverage to the MIR-FIR region through optimized broadband phase-matching capabilities utilizing a 3.9 µm broadband seed source with higher pulse energy (~1.45 mJ). The achieved 18% conversion efficiency for wavelengths exceeding 6000 nm is competitive with state-of-the-art ZnGeP_2_-based IP-DFG systems reporting efficiencies up to 9.5%^47^. Our angular-tuning experiment (*θ* = 41°–49°) confirms that AGSe crystals exhibit a phase-matching angle tolerance exceeding ±4°. This robustness is manifested in preserved spectral flatness (Δ < 10 dB) and energy stability (>0.7 mJ/pulse) across the MIR-FIR range, making it feasible to realize robust MIR-FIR white laser sources without strict alignment protocols. This performance positions the cascaded LN-AGSe module as a promising candidate for next-generation MIR-FIR light sources, particularly in applications requiring ultrabroadband coverage and energy-efficient operation.

### High brightness DUV-FIR full-spectrum white laser in a synergic dual-channel scheme

**Fig. 5:**
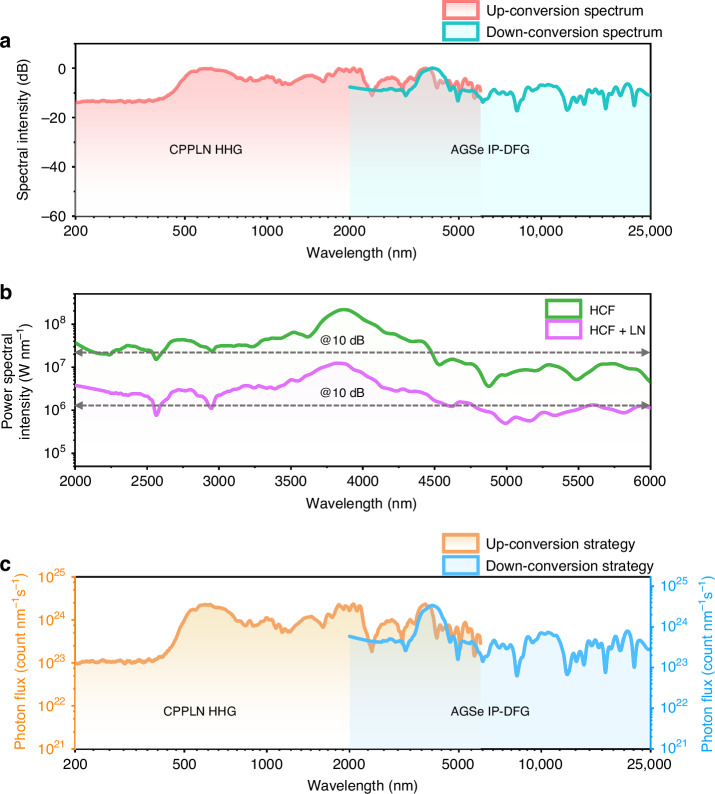
Intense high-flatness 7-octave DUV-FIR pulse white laser. **a** Synthesis of up-conversion and down-conversion white laser into DUV-FIR full-spectrum white laser. **b** The power spectral intensity of the MIR laser from the HCF setup and the HCF-LN module, respectively. A baseline of 10 dB is shown for the assessment of spectral flatness. **c** The photon flux spectral intensity of the generated full-spectrum white laser spanning from 200 to 25,000 nm with an output pulse energy of ~1 mJ and an estimated pulse duration of 1.8 ps

Figure 5a clearly demonstrates that a synergic dual-channel scheme of up-conversion HHG processes in CPPLN and down-conversion IP-DFG in AGSe can generate a mJ-level 7-octave full-spectrum white laser with a spectral range of 200–25,000 nm at 17 dB from an octave-spanning MIR seed pulse. The overall performance of the full-spectrum laser in terms of spectral intensity, bandwidth, flatness, and pulse energy has reached an unprecedented level. The big success of our nonlinear up-conversion and down-conversion synergy strategy mainly stems from the following aspects.

First, the intense MIR supercontinuum seed pulse laser derived from the upscaling home-built OPCPA system provides a solid pump foundation for high-efficiency nonlinear up and down conversion. As evident from Fig. 5b, the power spectral intensity of the first-stage MIR supercontinuum seed pulse spanning from 2000 to 6000 nm and measuring 3.62 mJ and 23 fs, generated by the HCF setup powered by a 7.12 mJ OPCPA upscaling system, reaches an impressive average peak power level of approximately 10^8^ W nm^−1^. Subsequently, a 2.02 mJ second-stage MIR supercontinuum seed pulse with a broadened pulse width of around 120 fs is produced by guiding the emerging pulse from the HCF into a 2-cm-length bare LN crystal and also up to a high level of 10^7^ W nm^−1^. Such a high pump energy level can effectively drive the nonlinear up- and down-conversion process to its full potential with a high conversion efficiency. Meanwhile, the broadband and smooth nature of the pump light source supplies ample frequency components to engage in a sequence of cascaded up- and down-conversion three-wave mixing processes. Figure 5b compares the HCF (green) and HCF-LN (purple) spectra using a 10 dB baseline, demonstrating that the engineered LN-based soliton and DW effects effectively compensate for the spectral imperfections in the HCF output spectrum, achieving a 3 dB shortwave elevation (2000–2500 nm) and 4 dB longwave enhancement (4500–6000 nm). This spectral rebalancing manifests as enhanced bandwidth utilization across the MIR range. Such a feature guarantees a broad-spectrum frequency conversion and offers greater flexibility in filling gaps within the down-conversion spectral curve, ultimately improving the flatness of the spectrum.

Second, excellent phase-matching capability is fully harnessed and works very well in both channels. The superior performance of broad QPM bands in the designed CPPLN sample enables efficient 2nd–12th HHG processes with an intense one-octave pump laser, utilizing flexible poling period modulation without the need for complex linear dispersion engineering. The designed AGSe exhibits a broad BPM bandwidth, low group velocity dispersion, robust angular tolerance, and excellent 2nd-NL performance upon an octave-spanning longer-wavelength MIR pump, enabling unprecedented spectral coverage and conversion efficiency in the MIR to FIR region. Third and finally, the CPPLN and AGSe crystals in the up-conversion and down-conversion channels are both bulk materials with millimeter-scale cross-sections. They can tolerate orders-of-magnitude higher laser pulse energies than microstructured fiber optic systems, thereby facilitating the production of intense lasers from the nonlinear material level. All these pivotal geometric, physical, and material properties in the nonlinear up-conversion/down-conversion two-channel synergy laser system work well together collectively and constructively and contribute to the successful production of a high-quality full-spectrum white laser.

As anticipated, this experimental full-spectrum white laser source demonstrates exceptional brightness in terms of photon flux spectral intensity, as illustrated in Fig. 5c. The photon flux spectral intensity reaches an impressive level of 10^23^~10^24^ count nm^−1^ s^−1^ among the whole DUV-FIR region, surpassing the maximum brightness achievable from current large-scale facilities, such as the third-generation synchrotrons, by 7–8 orders of magnitude^48^. Such exceptional single-pulse brightness guarantees both sufficient energy for nonlinear interactions and high photon counts for noise-immune measurements. As revealed clearly by the detailed comparative analyses made in Supplementary Note 6, the current full-spectrum white laser has made a big advancement upon previous representative schemes in several crucial performance metrics as pulse energy, spectral bandwidth, and spectral flatness, because of the innovation made in laser system architecture, nonlinear physical mechanism, crystal structural optimization, and pumping laser condition. This cutting-edge full-spectrum white laser opens the door to the development of a compact high-performance laser system suitable for various applications in ultrafast time-resolved spectroscopy and broadband imaging applications. Its brightness advantage over synchrotrons, combined with mJ-level energy scalability and tabletop portability, would greatly cater to the specific needs of basic science disciplines such as physics, chemistry, materials science, and biology, and provide an outstanding illumination source on a tabletop scale for these areas.

## Discussion

In summary, we have presented the creation of an intense DUV-FIR white laser with 200–25,000 nm bandwidth at -17 dB and ~1 mJ pulse energy by exploiting the synergic action of a high-efficiency nonlinear up-conversion module and down-conversion module upon an intense MIR seed pulse laser. The MIR seed pulse laser of 3.62 mJ pulse energy is achieved by sending an OPCPA pulse laser of 7.12 mJ pulse energy and 3.9 μm central wavelength through a Kr-gas-filled HCF. The up-conversion nonlinear module is a deliberately designed CPPLN nonlinear crystal supporting simultaneous broadband 2nd-NL 2nd–12th HHG upon the MIR supercontinuum seed laser to generate the shortest DUV wavelength down to 200 nm with a nearly 40% conversion efficiency. The down-conversion nonlinear module is composed of a bare LN crystal offering excellent 3rd-NL spectral broadening and a cascaded AGSe nonlinear crystal offering high-efficiency IP-DFG to generate a 2000–25,000 nm MIR-FIR laser with an overall conversion efficiency of ~18%.

An intense supercontinuum ultrafast pulse white laser with continuous DUV-FIR spectral coverage can open up a new arena of ultrafast and ultrabroadband laser spectroscopy with important applications to a wide variety of basic science and technology areas. The systematic spectroscopic applications of the DUV-FIR full-spectrum pulse white laser have clearly demonstrated its great power in deeply examining, exploring, and disclosing many spectroscopic features originating from various electronic transitions, molecular vibration, and lattice oscillation processes underlying a variety of matters and materials. This great power should be ascribed solely to the unprecedented performances of the developed white laser in terms of several critical indices as spectral intensity, bandwidth, flatness, pulse energy, duration, brightness, and coherence. In principle, this DUV-FIR white laser can monitor the spectroscopic response of these matters and materials simultaneously, provided that an advanced DUV-FIR spectrometer instrument system is built to join seamlessly with the DUV-FIR white laser system^24,49,50^. This system would offer unprecedented opportunities to study physical, chemical and biological processes over extreme spectral bandwidths and timescales, disclose the unknown mutual interaction rules and laws of multi-energy scale processes (e.g., electron-phonon interaction in solid), and monitor the spectroscopic response of a great variety of matters and materials in real time and in site under the influence by various factors such as temperature, electric field, magnetic field, concentration, and the presence of other matters and materials.

## Materials and methods

### Fabrication of CPPLN and AGSe samples

The CPPLN sample was fabricated using a room-temperature electric poling technique applied to a 2-mm-thick optical-grade *z*-cut LN crystal^24,37–39^. The process involved photoresist deposition on the +*Z* surface, lithographic patterning with designed chirped lattices, and selective exposure to a saturated lithium chloride electrolyte. Domain inversion was achieved by applying a 24 kV/mm electric field pulse across the protected and exposed regions of the crystal. The AGSe crystal was manufactured by Chengdu Dien Photoelectric Technology Co., Ltd. after we determined the optimal phase-matching regime and optimal phase-matching angle through difference-frequency phase-matching design.

### Spectral measurements

The full-spectrum white laser spanning DUV-UV-Vis-NIR-MIR-FIR bands (200–25,000 nm) was characterized using a multi-instrument approach. The DUV-UV-Vis range (200–1100 nm) was measured with an Ocean Optics HR 4000 spectrometer, while the Vis-NIR region (600–1700 nm) was analyzed using a Yokogawa AQ 6370D optical spectrum analyzer. For the NIR-MIR bands, an Ocean Optics NIR Quest was used to record 900–2500 nm spectra, and a FTIR spectrometer (Bruker Vertex 70) equipped with an MCT detector cooled by liquid nitrogen extends the spectral measurements to 2000–25,000 nm. Data calibration involved interpolating overlapping regions based on intensity standards, where these spectrometers consistently delivered accurate measurements was performed to finally obtain the full-spectrum profile.

## Supplementary information

Supplemental Material

## Data Availability

The data that support the findings of this study are available from the corresponding author on reasonable request.
