# Supplementary material for: mJ-level 7-octave ultraflat white laser encompassing 200–25,000 nm
Source: Light Sci Appl. 2026 Jan 20;15:72. doi: 10.1038/s41377-025-02142-z (PMC12816706; doi:10.1038/s41377-025-02142-z)
Supplement: Supplementary file 1 — Supplemental Material [file 41377_2025_2142_MOESM1_ESM.docx]

Supplementary Materials for

**mJ-level 7-octave ultraflat white laser encompassing 200-25000 nm**

Lihong Hong^1,2,3^, Renyu Feng^2^, Yuanyuan Liu^1^, Junming Liu^1^, Junyu Qian^2^, Yujie Peng^2^, Yuxin Leng^2^, Ruxin Li^2,*^, and Zhi-Yuan Li^1,4*^

^1^School of Physics and Optoelectronics, South China University of Technology, Guangzhou 510641, China

^2^State Key Laboratory of Ultra-intense Laser Science and Technology, Shanghai Institute of Optics and Fine Mechanics, Chinese Academy of Sciences, Shanghai 201800, China

^3^Guangdong Jingqi Laser Technology Corporation Limited, Dongguan 523808, China

^4^State Key Laboratory of Luminescent Materials and Devices, South China University of Technology, Guangzhou 510640, China

*Correspondence should be addressed to Zhi-Yuan Li: [phzyli@scut.edu.cn](mailto:phzyli@scut.edu.cn); Ruxin Li: ruxinli@mail.shcnc.ac.cn.

**This file includes:**

**Supplementary Text**

Supplementary Note 1 **MIR pump laser and visible-band white laser beam profile**

Supplementary Note 2 **MIR seed laser supercontinuum physics**

Supplementary Note 3 **Spectral and temporal properties of 2^nd^-12^th^ HHGs within CPPLN**

Supplementary Note 4 **CPPLN architecture optimization**

Supplementary Note 5 **Spectral and temporal properties of IP-DFG within AGSe**

Supplementary Note 6 **Our white laser performance in comparison with previous representative works**

**References 1-7**

**Supplementary Text**

**Supplementary Note 1 | MIR pump laser and visible-band white laser beam profile**

The spatial beam quality and temporal stability of our optical system are critical for achieving efficient and reproducible full-spectrum white laser. We have systematically evaluated the stability of our white light laser system at multiple levels. **Figure S1a** presents beam spot photographs from three key optical modules, captured using wavelength-appropriate detection methods. The MIR pump laser at 3900 nm exhibits a near-Gaussian spatial profile with excellent beam quality, as measured using a PyroCAM IV pyroelectric array camera with a resolution of 80 μm, optimized for MIR wavelengths (left column, **Fig. S1a**). The following HCF propagation further improves beam quality through mode-selective transmission, where the fundamental mode exhibits substantially higher transmission efficiency than higher-order modes under our coupling conditions, providing inherent spatial beam cleaning while enabling efficient spectral broadening (middle column, **Fig. S1a**). The final CPPLN module output demonstrates uniform spatial intensity distribution across the beam aperture, characterized using a conventional visible-light digital camera (Nikon D7200), maintaining excellent beam quality throughout the up-conversion process (right column, **Fig. S1a**). Notably, the output beam spots from OPCPA through HCF to CPPLN white light generation consistently maintain the original geometric shape and contour of the fundamental wave pump laser, demonstrating excellent beam profile preservation throughout the cascaded nonlinear optical conversion chain despite the dramatic spectral transformation from narrow-band MIR to ultrabroadband DUV-MIR coverage.

Regarding coherence properties, our system begins with a high-quality OPCPA pump coherent source, and the inherent nature of nonlinear optical processes preserves and transfers coherence characteristics, enabling the white light laser to achieve excellent temporal and spatial coherence. The MIR pump light retains sufficient temporal coherence to enable CaF_2_-based pulse compression even after HCF propagation. Critically, our entire nonlinear conversion architecture avoids plasma generation and other uncontrollable energy exchange mechanisms that could degrade laser coherence. While the cascaded up-conversion and down-conversion processes create complex temporal pulse waveforms, the fundamental coherence properties remain intact throughout the system. All these are in consistence with the nonlinear optical principle: Nonlinear frequency conversion maintains the coherence (both spatial and temporal coherence) of pump laser beam into signal laser beam because no energy exchange process is involved to destroy the coherence.


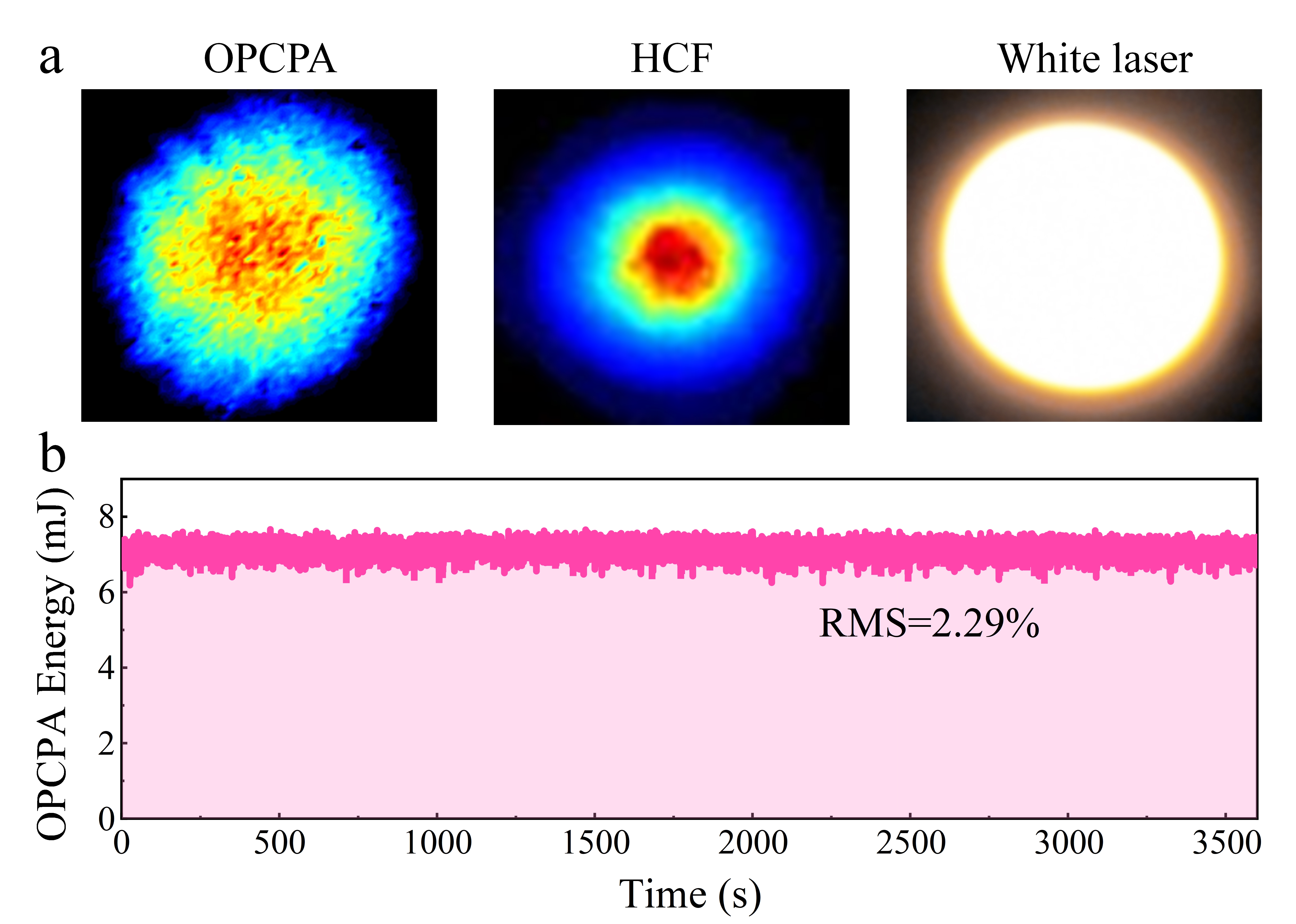


**Figure S1. a**, Beam spot photographs from three optical modules measured using wavelength-appropriate detection methods. The beam spot output from OPCPA (centered at 3900 nm) and HCF (covering 2000-6000 nm) modules are characterized using a pyroelectric array camera (PyroCAM IV) with a resolution of 80 µm. The CPPLN module (covering 200-6000 nm) is measured using a conventional visible-light digital camera (Nikon D7200). **b**, Long-term energy stability measurement of 3900 nm OPCPA pump laser pulse within 3600 s.

Long-term stability analysis reveals exceptional performance consistency of the 3900 nm OPCPA pump laser. As shown in **Fig. S1b**, energy fluctuations remain within ±2% root-mean-square (RMS) over a 3600-second measurement period, demonstrating the robust operation required for precision supercontinuum experiments. This temporal stability, combined with the high spatial beam quality and preserved coherence characteristics, ensures reproducible experimental conditions and reliable spectral broadening performance across extended measurement campaigns.

**Supplementary Note 2 | MIR seed laser supercontinuum physics**

To elucidate the fundamental physics governing multioctave MIR seed laser formation in the process of OPCPA pump laser pulse passing through the cascaded HCF and LN module, we perform comprehensive theoretical modeling and numerical simulations of the nonlinear pulse dynamics within the HCF system and subsequent LN crystal using experimental parameters to ensure direct comparison with observed phenomena.


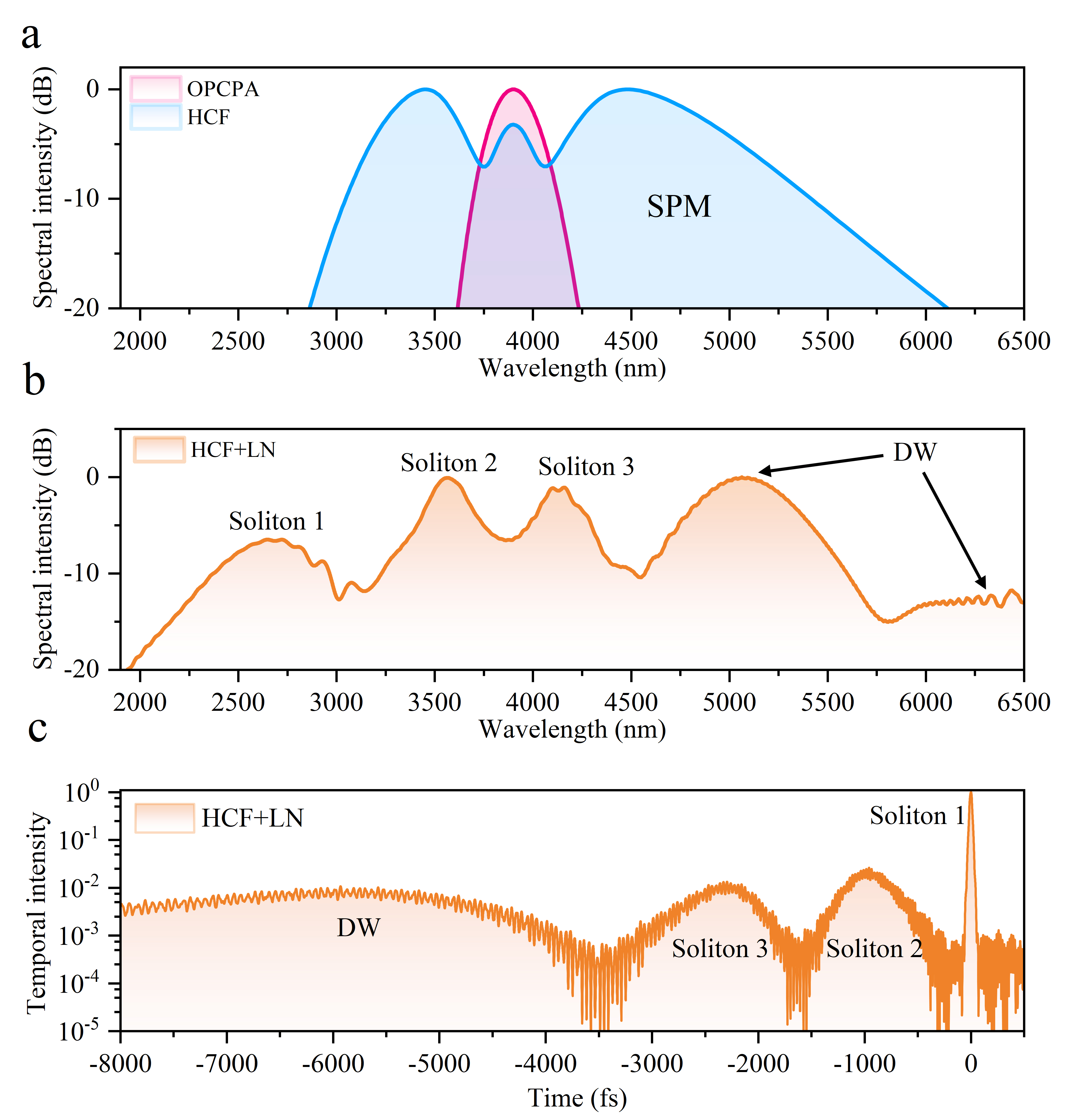


**Figure S2.** **a-b**, Numerical spectral simulations of MIR seed laser emitted from HCF and HCF-LN modules and the original spectral profile from the 3900 nm OPCPA setup, respectively. **c**, Temporal dynamics of solitons and DW within the HCF-LN module.

**(i) HCF spectral broadening.** A 3.9 μm pump pulse with 120 fs duration and 7.12 mJ pulse energy is launched into a 3 m Kr-filled HCF fiber maintained at 2.2 bar pressure. The temporal and spectral evolution is calculated using the split-step Fourier method to solve the generalized nonlinear Schrödinger equation^1-3^, incorporating both material dispersion of Kr gas and intensity-dependent refractive index effects. The pressure-dependent nonlinear coefficient is set to *n*_2_ = 2.7*p* × 10^-23^ m^2^ W^-1^, where *p* represents the Kr gas pressure (in unit of bar)^4-5^.

Dispersion analysis confirms that the Kr-filled HCF operates exclusively in the normal dispersion regime across the spectral range of interest. This dispersion characteristic ensures that SPM serves as the primary broadening mechanism, generating intensity-dependent frequency chirp that translates to spectral expansion upon propagation. The symmetric nature of SPM-induced broadening produces substantial bandwidth expansion spanning 2.87-6.1 μm at the 20 dB level (blue curve in **Fig. S2a**), with pronounced spectral extensions toward both the short- and long-wavelength regions compared with the initial OPCPA pump spectrum (magenta curve in **Fig. S2a**). Simulated spectral profiles exhibit excellent quantitative agreement with experimental measurement (cyan line in **Fig. 2e**, main text) in bandwidth, spectral shape, spectral peaks and conversion efficiency, confirming that our computational model captures the essential features of the experimental spectral evolution. Residual discrepancies between theoretical predictions and experimental observations can be attributed to higher-order nonlinear phenomena excluded from our simplified model, including pulse self-steepening, stochastic four-wave mixing processes, and competing nonlinear effects that introduce fine-scale spectral modulation beyond the dominant SPM contribution.

**(ii) Soliton dynamics and dispersive wave generation in LN crystal.** The spectrally broadened MIR pulse output from the HCF propagates into the bare LN crystal, triggering fundamentally different nonlinear dynamics. The LN crystal, operating in the anomalous dispersion regime for this injected MIR wavelength range, supports complex soliton formation accompanied by dispersion wave (DW). This secondary broadening process in LN crystal extends the 20 dB spectral bandwidth from 1.94 μm to 6.5 μm (orange curve in **Fig. S2b**), demonstrating the synergistic interplay between soliton and DW dynamics compared to the HCF output alone. Our theoretical framework quantitatively captures the experimental observations, with calculated spectral features showing remarkable consistency with the measured 4 dB flatness improvement and around 2000-6000 nm bandwidth extension. The spectral decomposition reveals three distinct soliton contributions: Soliton 1 dominates the spectral range from 2.0-5.75 μm through pronounced self-frequency shifting, while Solitons 2 and 3 occupy narrower bands at 3.20-3.83 μm and 3.80-4.46 μm, respectively, without observable frequency shifting. Concurrently, longer-wavelength DWs extend the long-wavelength coverage to 4.3-6.5 μm, completing the multioctave spectral architecture.

**Figure S2c** reveals the hierarchical temporal structure of these soliton structures alongside the DW components throughout the LN crystal. The dominant Soliton 1 maintains a coherent 26 fs full width at half maximum (FWHM) within a broader 140 fs temporal window, initially formed at 3.2 μm center wavelength before undergoing pronounced self-frequency shifting. This continuous redshift process in the anomalous dispersion regime enables its broad spectral coverage, with spectral evolution clearly tracked in **Fig. S2b**. Solitons 2 and 3 exhibit significantly lower energy densities with FWHM durations of 447 fs and 520 fs, respectively, maintaining fixed spectral positions centered at 3.57 μm and 4.16 μm. Despite their lower intensities, these secondary solitons contribute crucially to enhanced spectral flatness, improving the overall MIR supercontinuum uniformity.

DW occurs primarily driven by soliton self-frequency shifting and multi-soliton interactions within the LN crystal. These DW signals exhibit remarkably extended temporal profile spanning ~4000 fs (**Fig. S2c**) and inherently low intensity that is further reduced by material absorption at longer wavelengths (**Fig. S2b**). Despite these limitations, the DWs enhance both spectral breadth and flatness in the long-wavelength region of 4.3-6.5 μm, establishing the full multioctave MIR seed spectrum. The above discussions also indicate that theoretical and numerical analyses can become invaluable and reliable tool for disclosing some delicate features of this highly complicated nonlinear interacting system. In some situations, they are indispensable because they can offer supplemental detailed information that are hard to reach by contemporary experimental tools.

**Supplementary Note 3 | Spectral and temporal properties of 2^nd^-12^th^ HHGs within CPPLN**

In this section, we theoretically and numerically analyze full-spectrum white laser generation in the nonlinear up-conversion CPPLN module through comprehensive modeling of the complete harmonic generation sequence, spanning from the broadband MIR supercontinuum from HCF input to the 12^th^-HHG output in the DUV region. The CPPLN crystal supports 36 distinct three-wave mixing channels capable of generating 2^nd^-12^th^ harmonics through various combinations of frequency doubling and sum-frequency processes. Notice that all these 36 nonlinear processes have considerable efficiencies, so that they strongly couple with each other and no one can be simply omitted. This makes the full wave simulation of these nonlinear processes highly complicated and intractable even with contemporarily most advanced nonlinear optics theoretical and numerical tools. In this regard, for computational efficiency while preserving the essential nonlinear physics, we suffice ourselves to only focus on one representative pathway that demonstrates the fundamental cascaded frequency conversion mechanism. This pathway proceeds sequentially: the MIR supercontinuum pump undergoes SHG to produce the 2^nd^-HHG, which subsequently serves as the seed for 4^th^-HHG via another SHG process. The 4^th^-HHG then drives its own SHG to generate the 8^th^-HHG, while the final 12^th^-HHG emerges through sum-frequency generation between the 4^th^- and 8^th^- HHGs.


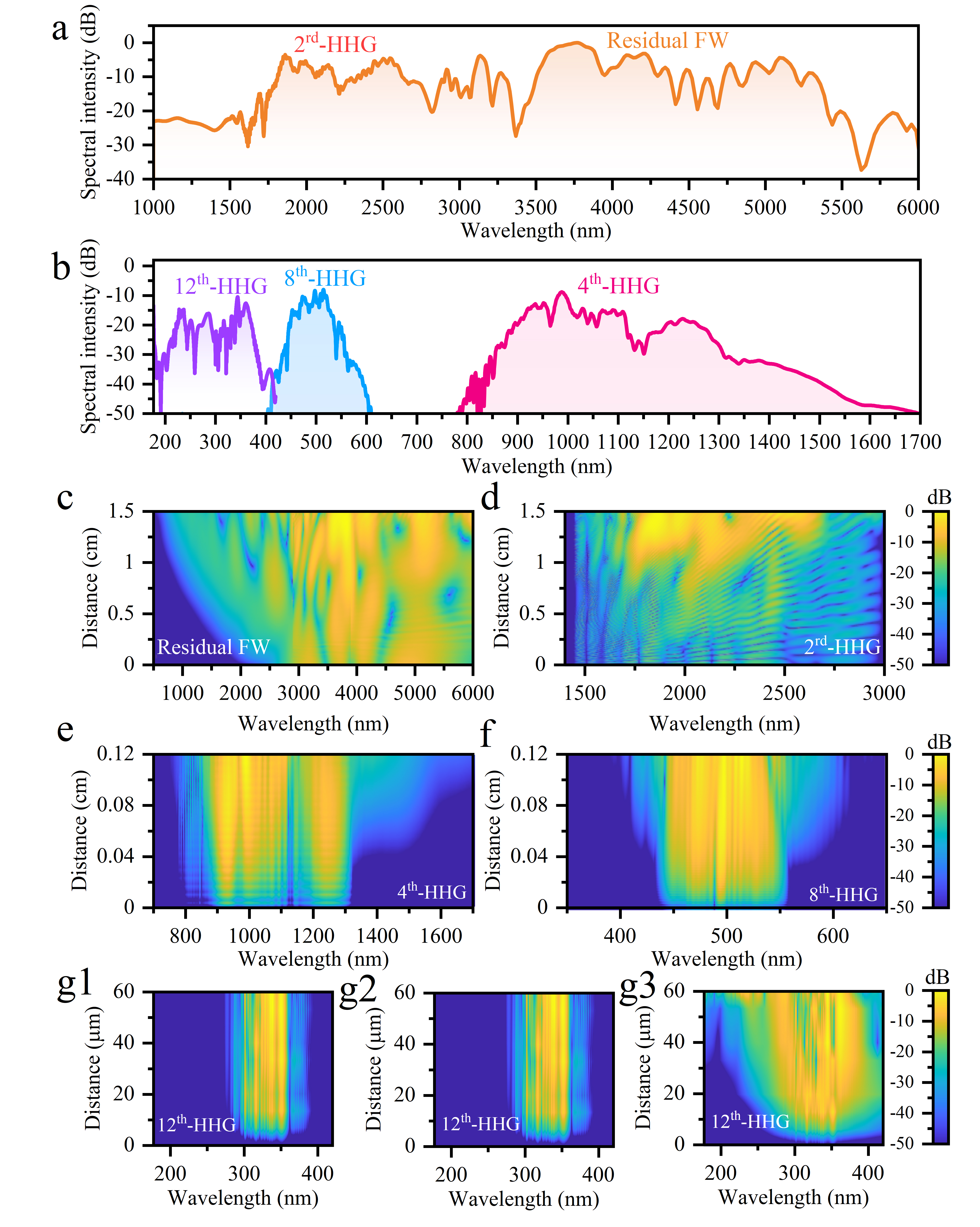


**Figure S3.** **a**, Simulated interconnected spectra of the generated 2^nd^ HHG and the residual FW at different transmission distances. **b,** Simulated spectra of 4^th^ HHG, 8^th^ HHG, and 12^th^ HHG output from the CPPLN sample under the action of 2^nd^-NL and 3^rd^-NL effects. **c-f**, Spatio-spectral maps of the remaining FW, 2^nd^-, 4^th^-, 8^th^-HHGs transporting into different distance of CPPLN crystal under the action of 2^nd^-NL and 3^rd^-NL effects. **g1-g3,** Spatio-spectral maps of 12^th^- HHG in different cases: **(g1)** 2^nd^-NL without considering LN materialabsorption, **(g2)** 2^nd^-NL considering LN material absorption, and **(g3)** 2^nd^-NL and 3^rd^-NL together with LN material absorption considered.

**Figure S3a-b** illustrates the overall spectral profile of the generated 2^nd^-, 4^th^-, 8^th^-, 12^th^-order HHGs and residual fundamental wave (FW) at different propagation distances through the CPPLN crystal. The spectrum exhibits distinct harmonic contributions (at -50 dB level): 12^th^-HHG peaks dominate the DUV region (200-380 nm), 8^th^-HHG covers the visible range (400-600 nm), 4^th^-HHG spans the NIR region (770-1700 nm), and 2^nd^-HHG extends across 1450-3000 nm with balanced spectral density. The residual FW occupies 3000-6000 nm with decreasing intensity at longer wavelengths. The complete spectral envelope demonstrates seamless continuity across all harmonic orders with minimal gaps, confirming effective cascaded frequency conversion from MIR to DUV wavelengths and validating the correctness of our architectural approach. Importantly, this simulation represents only one of the 36 possible harmonic generation channels, suggesting the actual experimental output should exhibit significantly broader spectral coverage and enhanced intensity than this single-pathway simulation (as the result in **Fig. 3a**).

**Figure S3c-g3** illustrates the spatio-spectral evolution of harmonic generation throughout the CPPLN crystal. Upon entering the chirped poling region, the FW undergoes rapid third-order nonlinear spectral broadening while simultaneously triggering SHG process. The residual FW (**Fig. S3c**) exhibits progressive spectral broadening from 1000-6000 nm over 1.5 cm propagation distance, with intensity redistribution showing initial concentration around 3000-4000 nm gradually expanding to both spectral edges. The 2^nd^-HHG (i.e. SHG) (**Fig. S3d**) develops over the same interaction length, spanning 1450-3000 nm with characteristic diagonal intensity patterns indicating efficient QPM assisted nonlinear conversion.

Higher-order harmonics emerge over increasingly shorter interaction lengths with concentrated spectral outputs. The 4^th^-HHG (**Fig. S3e**) forms rapidly within 0.12 cm, covering 770-1700 nm with peak intensity around 1000 nm. The 8^th^-HHG (**Fig. S3f**) generates over the same distance, producing visible light from 400-600 nm with maximum conversion in the blue region (430-550 nm). To elucidate the physics of 12^th^-HHG in the DUV, we systematically analyze the individual contributions of nonlinear processes and material absorption properties. We examine three scenarios: second-order nonlinearity (2^nd^-NL) alone without LN material absorption considered (**Fig. S3g1**), 2^nd^-NL with LN material absorption considered (**Fig. S3g2**), and the complete model incorporating 2^nd^-NL and third-order nonlinearity (3^rd^-NL) effects together with material absorption considered (**Fig. S3g3**). In **Fig. S3g1**, 12^th^ harmonic generation occurs uniformly across 280-360 nm throughout the 60-μm propagation distance. Comparison between **Fig. S3g1** and **Fig. S3g2** shows modest intensity reduction with similar spectral coverage, demonstrating that DUV material absorption, whilst present, remains manageable in the ultra-short interaction length. The **Fig. S3g2-g3** transition reveals a striking transformation: whilst **Fig. S3g2** shows minimal output in the 200-280 nm and 380-420 nm regions with weak generation elsewhere, **Fig. S3g3** demonstrates intense, localized generation concentrated precisely in these spectral ranges, particularly around 200-250 nm. This enhancement occurs exclusively in the final 10-15 μm of propagation, shifting from distributed generation to highly efficient short-wavelength conversion confined to the crystal terminus. This demonstrates the synergistic action of three mechanisms: The 2^nd^-NL establishes the fundamental harmonic conversion framework, the 3^rd^-NL processes drive spectral extension into the shorter-wavelength 200-250 nm DUV region, and ultra-short interaction lengths minimize material absorption whilst maintaining conversion efficiency through optimized chirped QPM. Overall, the generated 12^th^-HHG emerges within a 60 μm interaction zone, extending into the DUV (200-280 nm).

On the other hand, these spatio-spectral maps reveal the cascaded nature of harmonic generation, where the chirped poling structure of CPPLN enables sequential wavelength conversion through progressively decreasing poling periods. The shorter poling periods toward the crystal exit end provide larger phase-mismatch compensation required for higher-order harmonics (**Fig. 3d**), enabling 12^th^-HHG (down to 200 nm) to occur exclusively in the final propagation stages, where ultra-short interaction lengths minimize material absorption losses in the DUV region. This spatial distribution ensures optimal QPM conditions for each harmonic order to satisfy at specific crystal locations, enabling efficient multioctave frequency up-conversion from MIR to DUV through precisely engineered photon momentum compensation across the entire harmonic cascade.

**
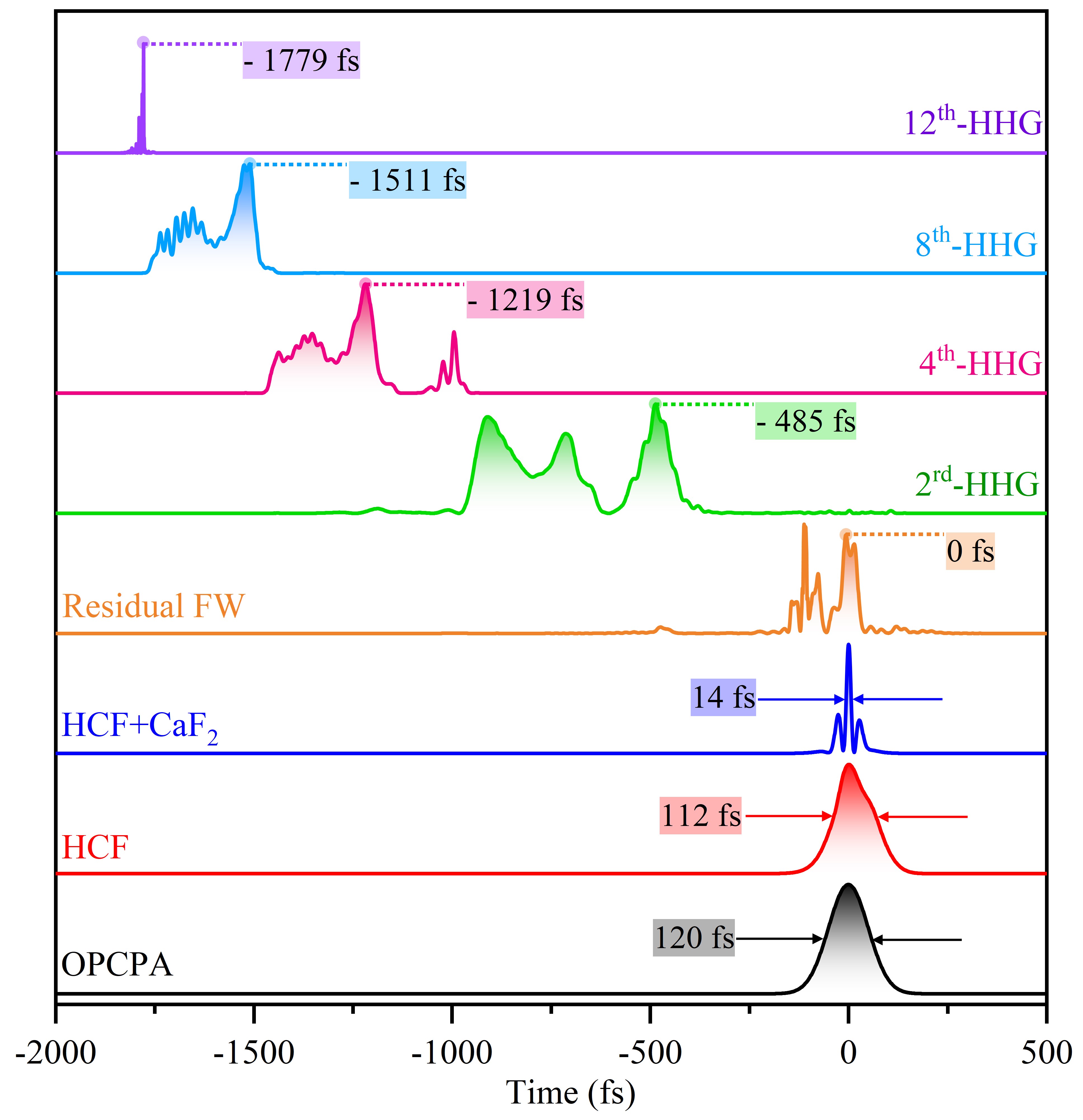
**

**Figure S4.** Simulated temporal evolution of laser pulse in the complete optical chain covering OPCPA pump passing through HCF, and subsequent cascaded nonlinear up-conversion processes within the 2-cm-length CPPLN crystal from FW to 2^nd^-HHG, 4^th^-HHG, 8^th^-HHG, and finally to 12^th^-HHG.

**Figure S4** reveals the temporal evolution throughout the above complete optical chain, tracking pulse characteristics from OPCPA pump passing through HCF, and subsequent cascaded nonlinear up-conversion processes within the CPPLN crystal from FW to 2^nd^-HHG, 4^th^-HHG, 8^th^-HHG, and finally to 12^th^-HHG. The initial 120 fs pump pulse at 3.9 μm undergoes modest compression to 112 fs after Kr-filled HCF propagation, followed by significant compression to 14 fs through the 2 mm CaF_2_ window, closely matching experimental observations. Upon entering the CPPLN crystal, the temporal structure becomes increasingly complex as high harmonic generation proceeds. The pulse temporal positions exhibit systematic shifts due to group velocity dispersion: the FW serves as the temporal reference (0 fs), while the 2^nd^-, 4^th^-, 8^th^- and 12^th^-HHGs are displaced to -485 fs, -1219 fs, -1511 fs, and -1779 fs, respectively. These temporal offsets, defined relative to the 3.9 μm pump center wavelength, agree excellently with group velocity dispersion calculations (477 fs, 718 fs, 1676 fs, and 1828 fs), validating the effectiveness and efficiency of our computational approach. The complete full-spectrum white laser output from the CPPLN crystal exhibits a total pulse duration of approximately 1.8 ps, encompassing all harmonic contributions covering DUV-MIR regions while maintaining the ultrafast characteristics essential for broadband applications. In other words, the CPPLN up-conversion module significantly extends the input MIR seed pulse with duration 14 fs to a duration of 1.8 ps (about 130 times expansion) for the DUV-MIR white laser pulse. The above numerical simulation also indicates that the group velocity dispersion between different harmonics from FW to 12^th^-HHG is the major reason for pulse temporal broadening. Hopefully this analysis could help to find a way for pulse compression for such a DUV-MIR all-spectrum white laser pulse.

**Supplementary Note 4 | CPPLN architecture optimization**

The newly-designed CPPLN crystal in this work achieves substantial performance improvements over our previous work (Ref. 39, main text) through systematic optimization of the B1 and B2 RLV bands governing 2^nd^, 3^rd^, and 4^th^ HHG via QPM. The critical advancement lies in significantly broadening the bandwidth of B1 band, so that it supports broader QPM bandwidth for 2^nd^ and 3^rd^ harmonics, and eliminating the spectral gap between B1 and B2 bands—a fundamental limitation of the earlier design with 41-24 μm poling periods which will hinder high efficiency generation of 4^th^ harmonics—through optimized poling periods spanning 37-15.35 μm (**Fig. S5**). More specially, the yellow highlighted regions illustrate the physical insight and principle underlying the crystal structure optimization: The new design eliminates the detrimental dip at 0.28-0.33 μm^-1^ present in the old B1-B2 band gap, achieving significantly broadened and flattened bandwidth B1 (0.14-0.43 μm^-1^). Although Fourier coefficients are reduced, the high-energy pumping compensates for this reduction, maintaining excellent conversion efficiency. The subsequent region B2 represents the extended stable operating range of 0.43-0.84 μm^-1^ without sharp decline. This structural optimization design successfully balances QPM bandwidth expansion with conversion efficiency, which supports a broader and smoother 4th-HHG.

The enhanced-performance microstructure incorporates extended negative domains of 6 μm beyond the positive domains, compared to the previous 4 μm extension, while implementing a higher chirp rate of 8.5 μm^-2^ versus the earlier 5.5 μm^-2^. This architectural optimization transforms the practical spectral response dramatically: the 4^th^-HHG maintains bandwidth coverage at 1000 nm while achieving a remarkable 10 dB improvement in spectral flatness, from 15 dB to an exceptional 5 dB dynamic range (**Fig. 3a** in the main text). The spectrally uniform and higher-efficiency 4^th^ HHG serves as the foundation for cascaded higher-order processes with improved efficiency, with its enhanced quality directly enabling efficient 8^th^-HHG and subsequent sum-frequency mixing between 4^th^- and 8^th^-HHGs to produce 12^th^-HHG. This process extends spectral coverage into the DUV region down to 200 nm, creating the ultra-flat white laser light spectrum essential for precision applications. The systematic CPPLN optimization ultimately demonstrates how microstructure engineering can overcome fundamental spectral limitations, establishing a robust platform for high-performance ultrabroadband coherent sources with simultaneous improvements in pulse energy, spectral coverage, and uniformity.


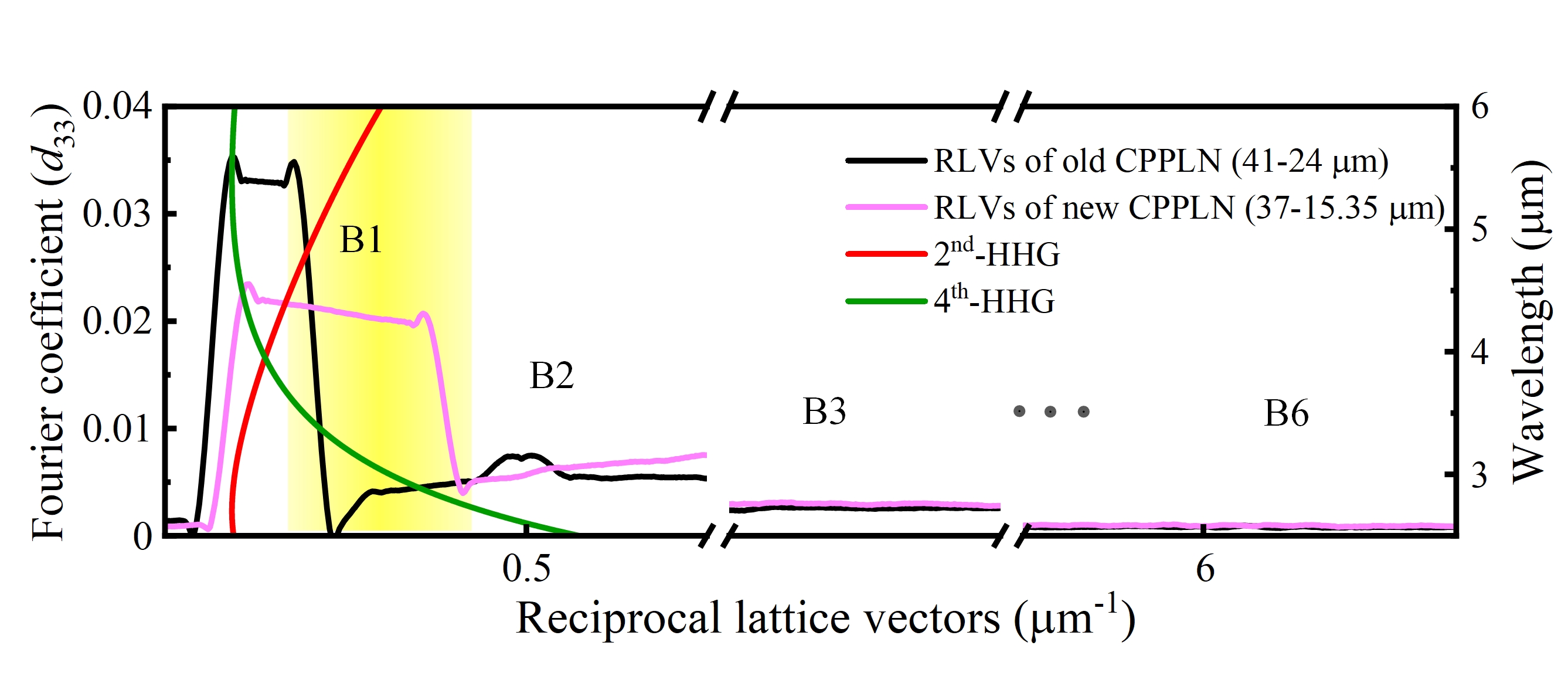


**Figure S5. Comparison of 4^th^-HHG QPM performance between the new CPPLN design (37-15.35 μm chirped periods, this work) and the old design (41-24 μm periods, Ref. 39).** Phase-mismatch curves for 2^nd^- and 4^th^-HHG processes in a homogeneous LN are shown alongside Fourier coefficient curves for B1-B6 QPM bands in both CPPLN crystals.

**Supplementary Note 5 | Spectral and temporal properties of IP-DFG within AGSe**

To comprehensively model the complete multioctave supercontinuum generation chain, we extend our theoretical and numerical analysis to include the down-conversion nonlinear module: the IP-DFG within the AGSe crystal by reference to the broadband nonlinear coupled wave theory^6-7^. The broadband LN output (**Fig. S2b**) serves dual roles as pump and signal beams, split equally to enable IP-DFG processes within the same pulse envelope. **Figure S6a** shows the complete IP-DFG spectral output from the HCF+LN+AGSe system spanning ~2000-30000 nm with intensity above -20 dB across most of the bandwidth. Notice that the output from AGSe crystal is the combination of input pump and signal pulse with the newly generated collinear idler pulse signal. The spectrum exhibits distinct plateaus: high intensity at 2000-6000 nm (above -10 dB), moderate intensity through 7500-15000 nm (-20 to -30 dB), and extended FIR coverage to 30000 nm. Pronounced shoulder peaks appear around 3000-5000 nm and 7500-12000 nm, with relatively smooth intensity transitions between different wavelength regions. Obviously, these simulated spectral characteristics, including plateau structure, intensity distribution, and peak positions, are consistent with experimental measurements (**Fig. 4a**). The IP-DFG process produces distinct temporal characteristics across the interacting waves (**Fig. S6b**). The pump and signal pulses maintain broad temporal profiles of ~600 fs (180 fs FWHM), while the generated idler pulse exhibits significant compression to 41 fs duration, completely covered by the residual pump pulse. This compression reflects efficient temporal focusing during difference frequency generation, where phase-matching conditions confine idler generation to optimal interaction regions. These simulation results indicate that the AGSe nonlinear down-conversion module only causes a modest pulse duration expansion upon the input MIR seed pulse, and this pulse duration is negligible in quantity compared with the case in the CPPLN nonlinear up-conversion module. In this regard, it can be assumed that when the DUV-MIR pulse output from the CPPLN module optimally overlaps with the MIR-FIR pulse output from the AGSe module, the synthetic DUV-MIR-FIR pulse will have a duration about 1.8 ps.

**Figure S6c** demonstrates energy transfer from pump and signal to idler waves during IP-DFG in AGSe. Both pump and signal waves initially exhibit concentrated energy around 2000-6000 nm that progressively depletes as propagation distance increases from 0 to 1 mm (the thickness of the AGSe crystal). The pump wave shows systematic energy extraction starting at 0.2 mm propagation, with depletion patterns expanding from 3000 nm to encompass 2500-5000 nm by 1 mm distance. The signal wave exhibits similar depletion characteristics, with energy reduction most pronounced in the 3000-4500 nm region after 0.6 mm propagation.


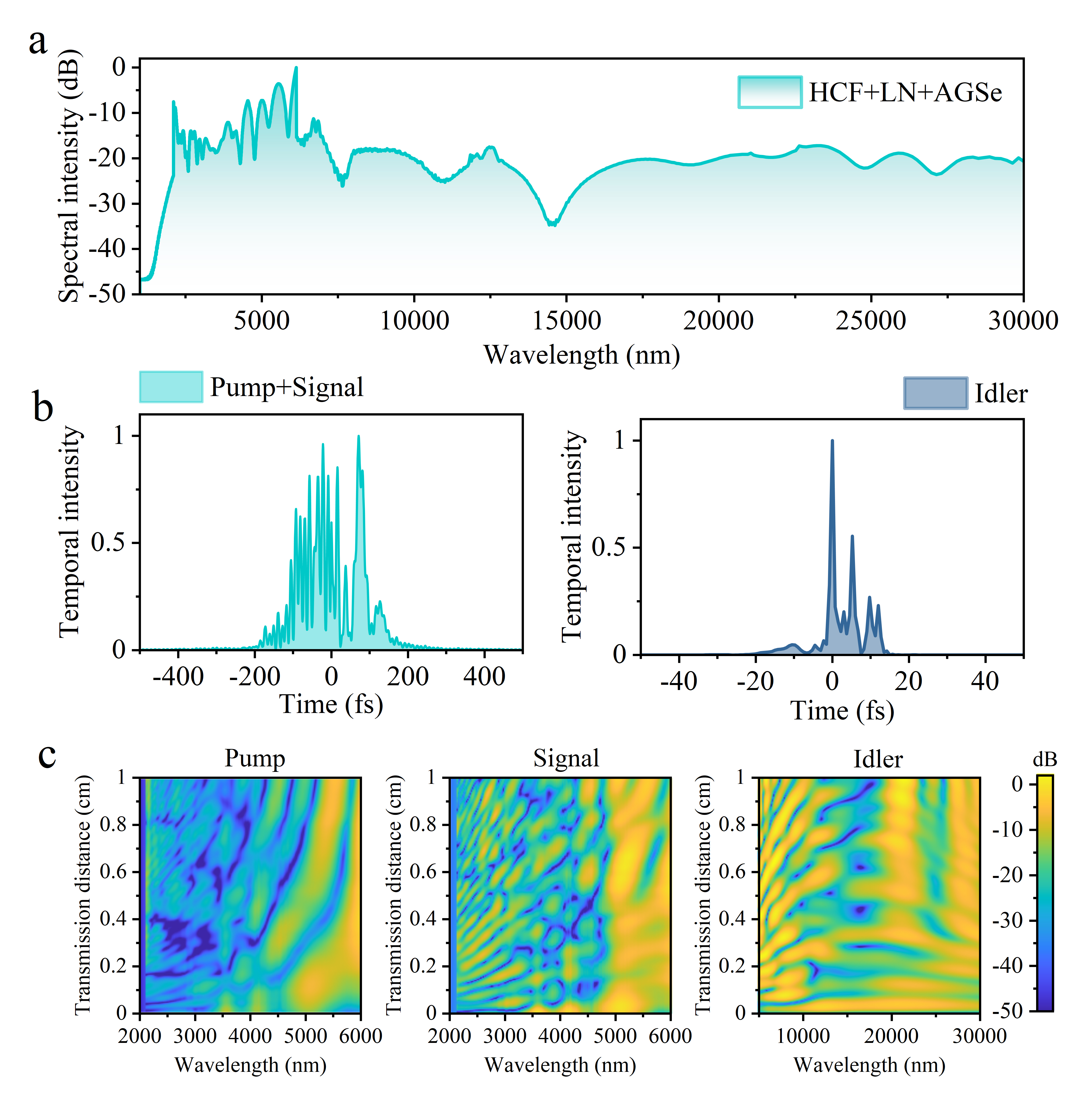


**Figure S6.** **a**, Simulated spectral profile of white laser pulse output from HCF-LN-AGSe nonlinear down-conversion module. **b**, Temporal profiles and **c**, spatial transmission volution of the pump, signal, and idler laser output from AGSe.

The idler generation directly reflects this energy depletion. Diagonal intensity patterns emerge at 0.2 mm propagation distance, initially generating wavelengths around 8000-12000 nm before extending to 30000 nm at full crystal length. The highest idler intensity occurs around 10000-15000 nm, coinciding with the maximum pump-signal depletion zones. Energy transfer proceeds wavelength-selectively: shorter pump-signal combinations (2000-3000 nm) deplete first, producing idler wavelengths around 5000-10000 nm, while longer combinations (4000-5000 nm) convert progressively to generate 15000-25000 nm idler frequencies. This spatially distributed energy transfer mechanism maximizes the conversion efficiency to 14.16% by enabling multiple simultaneous pump-signal combinations to feed energy into the idler wave. The broadband spectral depletion across the input beam ensures comprehensive utilization of available energy while creating continuous MIR-FIR coverage through multiple parallel wavelength-selective conversion channels. It is worth noting that the simulation predicts strong spectral signals extending to 25000-30000 nm, which currently cannot be measured in our experimental setup due to detector limitations in the FIR region. This confirms the potential of our IP-DFG approach to generate even broader FIR coverage. The spatio-spectral evolution simulation results displayed in **Fig. S6c** clearly show the spectral contribution of individual collinear pump pulse, signal pulse, and idler pulse to the finally output synthetic MIR-FIR pulse from the AGSe crystal.

**Supplementary Note 6 | Our white laser performance in comparison with previous representative works**

In this section, **we provide a systematic comparison among our work and two key representative references in ultrabroadband light source development: Elu et al. (Ref. 23: Seven-octave high-brightness and carrier-envelope-phase-stable light source. Nat. Photonics 15, 277-280 (2021)) and our previous work (Ref. 39: Intense ultraviolet-visible-infrared full-spectrum laser. Light Sci. Appl. 12, 199 (2023)).** The comparative analysis reveals fundamental distinctions that establish the significant advancement of our approach. Our dual-module cascaded system, integrating both up-conversion and down-conversion stages, represents a paradigmatic shift from previous ultrabroadband light source architectures through the synergistic integration of second-order and third-order nonlinear processes in high-energy strong-nonlinear regime.

The following presents a detailed comparison with Elu et al. (Ref. 23) and our previous work (Ref. 39) from several aspects.

**(1) Fundamental architectural innovation**

Our system employs a fundamentally different dual-module cascaded approach that addresses the limitations of previous methods:

**(i) Elu et al. system (Ref. 23)**: Uses microstructure anti-resonant reflecting photonic crystal (ARR-PCF) with soliton self-compression and dispersive wave generation for shorter wavelengths, combined with single-crystal IP-DFG for longer wavelengths. Although we believe this work has represented the best overall performance in supercontinuum white laser before our current work, this approach yields limited pulse energy (0.45 μJ) with poor spectral flatness measurable only at -70 dB level. The weakness can be ascribed to the fundamental constrain by the tiny modal area of the microstructure fiber, consequent weak pump pulse energy (17.5 μJ), and thus poor nonlinear frequency conversion efficiency to various spectral regimes.

**(ii) Our previous work (Ref. 39)**: Employs a simple single CPPLN approach with 3.3 mJ pump, capable of only 2^nd^-10^th^ HHG covering 300-5000 nm at -25 dB, but lacks any down-conversion module, significantly limiting spectral extension capabilities.

**(iii) This work**: Our system employs a cascaded dual-module approach that seamlessly integrates large-aperture HCF with bulk crystals frequency conversion. This architecture implements: (i) a comprehensive large-aperture HCF creating an octave-spanning mid-IR seed through SPM, (ii) a custom-designed CPPLN crystal enabling simultaneous 2^nd^-12^th^ harmonics extending to 200 nm in the DUV region, and (iii) a dedicated down-conversion module combining bare LN with cascaded AGSe crystals extending the spectrum to 25000 nm.

**(2) Physical principle innovation**

Our system operates at significantly higher pulse energy levels (5-10 millijoule-class) compared to previous work, dramatically enhancing nonlinear optical interactions and driving the processes into highly efficient regimes. Unlike conventional approaches relying on single-order nonlinear (mostly 3^rd^-NL) processes, our dual-module architecture uniquely integrates 2^nd^-NL and 3^rd^-NL in a coordinated manner throughout both up-conversion and down-conversion stages. This combination of synergistic nonlinearity integration and enhanced pulse energy is critical for achieving the extraordinary three-high white laser (high pulse energy, broad spectral coverage, and high spectral flatness), fundamentally differentiating our methodology from prior studies.

**(3) Performance breakthrough**

This architectural innovation enables substantial performance improvements across all key metrics, as displayed in **Table S1**:

**Table S1. Performance comparison of ultrabroadband white laser generation.**

| **Performance Metric** | **This Work** | **Elu et al. (Ref.23)** | **Our Previous Work (Ref. 39)** |
| --- | --- | --- | --- |
| Pulse energy | ~1 mJ | 0.45 μJ | 0.54 mJ |
| Initial pump energy | 7.12 mJ | 17.5 μJ | 3.3 mJ |
| Up-conversion module | 2^nd^-NL HHG~40% | 3^rd^-NL spectral broadening~80% | 2^nd^-NL HHG ~30% |
| Down-conversion module | IP-DFG~18% | IP-DFG ~2% | None |
| Spectral coverage | 200-25000 nm  @-17dB | 340-40000 nm  @-70dB | 300-5000 nm  @-25dB |

The three orders of magnitude improvement in pulse energy of our current work (~1 mJ) compared to Elu et al. (~0.45 μJ), combined with the dramatic 53 dB enhancement in spectral flatness (-17dB versus -70 dB), represents a quantum leap from proof-of-concept demonstrations to practical implementation. The achieved photon flux density of 10^23^-10^24^ count nm^-1^s^-1^ surpasses third-generation synchrotron facilities by 7-8 orders of magnitude, establishing new benchmarks for ultrabroadband laser light sources. This performance breakthrough stems from operating at 10 millijoule-class pulse energies that drive nonlinear interactions into highly efficient regimes previously unattainable in ultrabroadband supercontinuum generation.

**(4) Technical advantages and future scalability**

Our large-aperture HCF and bulk crystals architecture offers several critical advantages:

**(i) Energy handling capability**: Different from microstructure-limited design, our large-aperture HCF and bulk crystals architecture can handle much higher pulse energies without damage.

**(ii) Conversion efficiency**: The synergistic integration of second-order and third-order nonlinear processes in our dual-module architecture harnesses intense laser pumping to drive enhanced multichannel nonlinear optical responses, achieving unprecedented conversion efficiencies.

**(iii) Scalability potential**: Performance can be further enhanced by simply increasing the initial OPCPA energy, representing a clear pathway for future improvements.

**(iv) Spectral balance**: Our dual-module up-down conversion approach provides more balanced coverage across both short and long wavelengths, crucial for comprehensive spectroscopic applications.

For more clarity and brevity, we summarize the above comparisons in the following compact table, **Table S2**.

**Table S2. Comparative analysis of system architecture, physical mechanisms, and performance metrics for ultrabroadband light sources.**

| **Category** | **Parameter** | **This work** | **Elu et al. (Ref. 23)** | **Our previous work (Ref. 39)** |
| --- | --- | --- | --- | --- |
| **System architecture** | **Primary design** | Up conversion/ down conversion dual-module cascaded system | Microstructure fiber + IP-DFG | Single CPPLN module |
|  | **Spectral broadening** | Large-aperture Kr-filled HCF | Microstructure ARR-PCF | Large-aperture Kr-filled HCF |
|  | **Up-conversion module** | CPPLN  (2^nd^-12^th^ harmonics) | Soliton self-compression + DW | CPPLN  (2^nd^-10^th^ harmonics) |
|  | **Down-conversion module** | Cascaded LN-AGSe | Single-crystal ZGP/GaSe/BGGSe | None |
|  | **Pump configuration** | 7.12 mJ | 17.5 μJ | 3.3 mJ |
| **Physical mechanisms** | **Nonlinear order** | Synergistic 2^nd^-NL + 3^rd^-NL | Single 3^rd^-NL or 2^nd^-NL | Synergistic 2^nd^-NL + 3^rd^-NL |
|  | **Up-conversion process** | 2^nd^-NL 2^nd^-12^th^ HHG | 3^rd^-NL soliton + DW | 2^nd^-NL 2^nd^-10^th^ HHG |
|  | **Down-conversion process** | 3^rd^-NL spectral broadening + 2^nd^-NL IP-DFG | 2^nd^-NL IP-DFG | None |
|  | **Energy scaling** | High-energy nonlinear regime | Low-energy limitation | Moderate energy |
| **Performance metrics** | **Pulse energy** | ~1 mJ | 0.45 μJ | 0.54 mJ |
|  | **Spectral coverage** | 200-25000 nm | 340-40000 nm | 300-5000 nm |
|  | **Spectral flatness** | -17 dB | -70 dB | -25 dB |
|  | **Up-conversion efficiency** | ~40% | ~80% (3^rd^-NL spectral broadening) | ~30% |
|  | **Down-conversion efficiency** | ~18% | ~2% | None |
| **Technical advantages** | **Energy scalability** | High  (large-aperture) | Low  (microstructure-  limited) | Moderate  (damage threshold) |
|  | **Spectral uniformity** | Excellent  (-17 dB) | Poor  (-70 dB) | Moderate  (-25 dB) |
|  | **System complexity** | Moderate  (dual-module bulk crystals) | High  (microstructure fiber) | Low  (single-module bulk crystal) |
|  | **Practical applicability** | High  (>5mJ-class) | Limited  (sub-μJ) | Moderate  (<5mJ-class) |
|  | **Future enhancement** | Excellent  (OPCPA scaling) | Constrained  (modal limit) | Limited  (single-stage) |

Overall, we believe that our current work has presented a systematic approach to high-energy ultrabroadband white laser generation by developing a comprehensive system that synergistically combines high-efficiency up-conversion and down-conversion modules through coordinated second-order and third-order nonlinear processes acting on an intense MIR seed pulse. The key advantage of our current work compared with previous representative excellent works as Ref. 23 and Ref. 39 is the systematic integration of laser system architecture, nonlinear physical mechanism, crystal structural optimization, and pumping laser condition to enable high-quality ultrabroadband white laser generation. The coordinated integration of second-order and third-order nonlinearities throughout both up-conversion and down-conversion stages enables simultaneous optimization of harmonic generation, spectral broadening, and IP-DFG processes. Unlike microstructure-limited approaches that face fundamental scaling constraints, the large-aperture architecture provides clear pathways for further performance enhancement through OPCPA energy scaling.

Such an unprecedented intense, broadband, uniform and ultrafast white laser can greatly enhance the power of current laser spectroscopy, and find straightforward potential application in a wide variety of science and technology areas in optics, physics, chemistry, materials science, biology and medical sciences, and also information and environment sciences.

**References**

1. Agrawal, G. P. Nonlinear Fiber Optics. 5th edn. (Amsterdam: Elsevier, 2013).
2. Bache, M., Moses, J. & Wise, F. W. Scaling laws for soliton pulse compression by

cascaded quadratic nonlinearities. *Journal of the Optical Society of America B* **24**, 2752-2762 (2007).

1. Hong, L. H., Liu, Y. Y. & Li, Z. Y. Synergic action of linear dispersion, second-order nonlinearity, and third-order nonlinearity in shaping the spectral profile of a femtosecond pulse transporting in a lithium niobate crystal. *Photonics Research* **12**, 774-783 (2024)
2. Lehmeier, H. J., Leupacher, W. & Penzkofer, A. Nonresonant third order hyperpolarizability of rare gases and N_2_ determined by third harmonic generation. *Optics Communications* **56**, 67-72 (1985).
3. Ettoumi, W., Petit, Y., Kasparian, J. & Wolf, J. P. Generalized Miller formulae. *Optics Express* **18**, 6613-6620 (2010).
4. Hu, C. Y. et al. Theoretical solution to second-harmonic generation of ultrashort laser pulse. *Journal of Applied Physics* **122**, 243105 (2017).
5. Hong, L. H. et al. Spatial-temporal evolution of ultrashort laser pulse second harmonic generation in *β*-barium borate (*β*-BBO) crystal. *Journal of Applied Physics* **129**, 233102 (2021).
